# Supplementary material for: A family of archaeal hibernation factors that bind in tandem and protect ribosomes in dormant cells
Source: Nat Commun. 2026 Jun 25;17:8083. doi: 10.1038/s41467-026-74727-0 (PMC13454478; doi:10.1038/s41467-026-74727-0)
Supplement: Supplementary file 1 [file 41467_2026_74727_MOESM13_ESM.pdf]

nature portfolio

Corresponding author(s): Dipti D. Nayak
Jamie H. D. Cate

Last updated by author(s): Jan 20, 2026

Reporting Summary

Nature Portfolio wishes to improve the reproducibility of the work that we publish. This form provides structure for consistency and transparency in reporting. For further information on Nature Portfolio policies, see our Editorial Policies and the Editorial Policy Checklist.

Statistics

For all statistical analyses, confirm that the following items are present in the figure legend, table legend, main text, or Methods section.

n/a	Confirmed	
[ ]	[x]	The exact sample size (*n*) for each experimental group/condition, given as a discrete number and unit of measurement
[ ]	[x]	A statement on whether measurements were taken from distinct samples or whether the same sample was measured repeatedly
[ ]	[x]	The statistical test(s) used AND whether they are one- or two-sided<br>*Only common tests should be described solely by name; describe more complex techniques in the Methods section.*
[x]	[ ]	A description of all covariates tested
[ ]	[x]	A description of any assumptions or corrections, such as tests of normality and adjustment for multiple comparisons
[ ]	[x]	A full description of the statistical parameters including central tendency (e.g. means) or other basic estimates (e.g. regression coefficient) AND variation (e.g. standard deviation) or associated estimates of uncertainty (e.g. confidence intervals)
[ ]	[x]	For null hypothesis testing, the test statistic (e.g. *F*, *t*, *r*) with confidence intervals, effect sizes, degrees of freedom and *P* value noted<br>*Give P values as exact values whenever suitable.*
[x]	[ ]	For Bayesian analysis, information on the choice of priors and Markov chain Monte Carlo settings
[x]	[ ]	For hierarchical and complex designs, identification of the appropriate level for tests and full reporting of outcomes
[x]	[ ]	Estimates of effect sizes (e.g. Cohen's *d*, Pearson's *r*), indicating how they were calculated

Our web collection on statistics for biologists contains articles on many of the points above.

Software and code

Policy information about availability of computer code

Data collection	Serial EM 4.1.22
Data analysis	Bioinformatics:<br>HMMER 3.4, mafft 7.525, iTOL v6<br>Cryo-EM:<br>Relion 4, cryoSPARC 4<br>Modeling:<br>PHENIX 1.20.1, COOT 0.9.8.95, ChimeraX 1.10.1, ModelAngelo 1.0, AlphaFold 3<br>RNA sequencing:<br>Geneious Prime v2025.2.2, Bowtie2 v2.3.2, Samtools v1.6

For manuscripts utilizing custom algorithms or software that are central to the research but not yet described in published literature, software must be made available to editors and reviewers. We strongly encourage code deposition in a community repository (e.g. GitHub). See the Nature Portfolio guidelines for submitting code & software for further information.

Data

Policy information about availability of data

All manuscripts must include a data availability statement. This statement should provide the following information, where applicable:

- Accession codes, unique identifiers, or web links for publicly available datasets
- A description of any restrictions on data availability
- For clinical datasets or third party data, please ensure that the statement adheres to our policy

Ribosome coordinates have been deposited in the Protein Data Bank for the M. acetivorans 70S-SriA-SriB complex (9ZNF), 70S-SriD complex (9ZNG), and 70S-SriC-SriD complex (9ZNI). Cryo-EM maps have been deposited in the Electron Microscopy Data Bank for the 70S-SriA-SriB complex (EMD-74446, EMD-74841 EMD-74851, EMD-74853, and EMD-74854), 70S-SriD complex (EMD-74447, EMD-74982, EMD-74984, and EMD-74985), and 70S-SriC-SriD (EMD-74448, EMD-74988, EMD-74989, EMD-74990, and EMD-74992). Previously published ribosome models (PDB IDs 8QOA and 8EMM) were used in this study. Raw WGS and RNA-seq reads were deposited in the Sequencing Reads Archive (SRA) under Bioproject number PRJNA1468727. Previously published transcriptomic datasets we analyzed can be found in the SRA under Bioproject numbers PRJNA1107246, PRJNA823372, and PRJNA1292878. Source data are provided with this paper.

Research involving human participants, their data, or biological material

Policy information about studies with human participants or human data. See also policy information about sex, gender (identity/presentation), and sexual orientation and race, ethnicity and racism.

Reporting on sex and gender	N/A
Reporting on race, ethnicity, or other socially relevant groupings	N/A
Population characteristics	N/A
Recruitment	N/A
Ethics oversight	N/A

Note that full information on the approval of the study protocol must also be provided in the manuscript.

Field-specific reporting

Please select the one below that is the best fit for your research. If you are not sure, read the appropriate sections before making your selection.

[x] Life sciences [ ] Behavioural & social sciences [ ] Ecological, evolutionary & environmental sciences

For a reference copy of the document with all sections, see nature.com/documents/nr-reporting-summary-flat.pdf

Life sciences study design

All studies must disclose on these points even when the disclosure is negative.

Sample size	No sample size calculations were conducted. Triplicate independent readings were taken of in vitro translation and cell growth assays to measure the mean and standard deviation.
Data exclusions	no data were excluded from analyses
Replication	Triplicate replicates for all biochemical experiments were conducted. All experimental replicates followed the same data trends.
Randomization	This study did not involve system (biochemical and structural studies) that require randomization.
Blinding	Blinding was not used in this study. Data collected from structural and biochemical experiments were not subjected to investigator bias.

Reporting for specific materials, systems and methods

We require information from authors about some types of materials, experimental systems and methods used in many studies. Here, indicate whether each material, system or method listed is relevant to your study. If you are not sure if a list item applies to your research, read the appropriate section before selecting a response.

Materials & experimental systems

n/a	Involved in the study
☒	☐ Antibodies
☒	☐ Eukaryotic cell lines
☒	☐ Palaeontology and archaeology
☒	☐ Animals and other organisms
☒	☐ Clinical data
☒	☐ Dual use research of concern
☒	☐ Plants

Methods

n/a	Involved in the study
☒	☐ ChIP-seq
☒	☐ Flow cytometry
☒	☐ MRI-based neuroimaging

Plants

Seed stocks	*Report on the source of all seed stocks or other plant material used. If applicable, state the seed stock centre and catalogue number. If plant specimens were collected from the field, describe the collection location, date and sampling procedures.*
Novel plant genotypes	*Describe the methods by which all novel plant genotypes were produced. This includes those generated by transgenic approaches, gene editing, chemical/radiation-based mutagenesis and hybridization. For transgenic lines, describe the transformation method, the number of independent lines analyzed and the generation upon which experiments were performed. For gene-edited lines, describe the editor used, the endogenous sequence targeted for editing, the targeting guide RNA sequence (if applicable) and how the editor was applied.*
Authentication	*Describe any authentication procedures for each seed stock used or novel genotype generated. Describe any experiments used to assess the effect of a mutation and, where applicable, how potential secondary effects (e.g. second site T-DNA insertions, mosiacism, off-target gene editing) were examined.*
