# Supplementary material for: A family of archaeal hibernation factors that bind in tandem and protect ribosomes in dormant cells
Source: Nat Commun. 2026 Jun 25;17:8083. doi: 10.1038/s41467-026-74727-0 (PMC13454478; doi:10.1038/s41467-026-74727-0)
Supplement: Supplementary file 2 [file 41467_2026_74727_MOESM14_ESM.pdf]

nature portfolio

Peer Review File

A family of archaeal hibernation factors that bind in tandem and protect ribosomes in dormant cells

Corresponding Author: Professor Jamie Cate

This file contains all reviewer reports in order by version, followed by all author rebuttals in order by version.

Version 0:

Reviewer comments:

Reviewer #1

(Remarks to the Author)
The manuscript by Nissley, Williams et al., entitled " A family of archaeal hibernation factors that bind in tandem and protect ribosomes in dormant cells", presents a comprehensive study of a set of ribosome hibernation factors (SriA–SriD) in the methanogenic archaeon Methanosarcina acetivorans. By combining transcriptomics, genetics, in vitro translation assays, cryo-EM, and phylogenetic analysis, the authors propose that Sri proteins repress translation, protect ribosomes during prolonged stationary phase, and bind ribosomes as paired factors at two distinct functional sites. The cryo-EM reconstructions are of excellent quality, and the phylogenetic analysis convincingly demonstrates that Sri homologs are broadly distributed across archaea. Overall, the work is strong and represents a very valuable contribution to the field of archaeal ribosome biology. However, several aspects of the functional analysis and mechanistic interpretation require clarification or additional experimental support.

Major comments:

1. The primary in vivo phenotype observed in the ΔsriABCD strain is an extended lag phase (28 days post-exponential growth). However, M. acetivorans is known to encode additional ribosome hibernation systems, including MRDF and potentially Hib homologs, which could compensate for the loss of Sri proteins. This potential functional redundancy complicates the interpretation of the phenotype. The authors should therefore discuss more explicitly how the presence of alternative hibernation factors might buffer the effects of the sri deletion.

2. All functional conclusions rely solely on the quadruple ΔsriABCD knockout. Because SriA/B associate with the large ribosomal subunit, whereas SriC/D bind the small subunit, and because these factors display distinct in vitro activities, the use of only a combined deletion makes it difficult to assign specific roles to each module. If possible, generating and analyzing ΔsriAB and ΔsriCD strains would potentially allow separation of LSU- and SSU-specific effects and provide a more mechanistically precise interpretation.

3. Translation inhibition is evaluated at only a single Sri concentration (6 μM), and no estimate of the intracellular abundance of Sri proteins is provided. Without concentration-dependent curves in the in vitro translation assays, it is difficult to judge whether the observed inhibition reflects physiologically relevant interactions. Performing concentration-dependent or titration experiments would greatly strengthen the interpretation and biological relevance of the in vitro translation results.

4. The cryo-EM data indicate that SriC occupancy is low, even when SriC and SriD are supplied at high concentrations. If SriC has intrinsically low affinity for the ribosome, this raises questions about its stability and functional relevance in vivo. The authors should therefore discuss whether SriC binding is subject to regulation, for example through post-translational modifications or nucleotide/cofactor dependence.

5. The authors argue that circularization of the 23S rRNA protects the LSU from exonucleolytic degradation. However, they also report a 21% reduction in domain IV coverage in the ΔsriABCD strain due to endonucleolytic cleavage. The authors should clarify in the manuscript whether SriA/SriB binding overlaps with or shields this region and whether the degradation site lies near their binding site, to better reconcile LSU protection with the observed domain IV degradation.

Minor comments:

1. The cryo-EM analysis identifies two coexisting ribosome populations: one bound by SriA/B and the other by SriD, each associated with a distinct small subunit rotational state. This is an intriguing observation, as it implies the presence of functionally distinct hibernated ribosome states. However, the manuscript does not explore the implications of this finding. The authors should discuss what conditions might determine the distribution between these states.

2. The proposed role of CBS domains in nucleotide sensing is also an important aspect. Given that CBS domains typically bind adenosine nucleotides and that sri expression is linked to cellular energy status, it would be useful to indicate whether the Sri CBS domains contain adenosine-binding motifs. Clarifying the structural features of these domains would strengthen the mechanistic discussion.

In summary, the study by Nissley, Williams et al. represents a significant contribution by providing a structural and evolutionary framework for archaeal ribosome hibernation. The high-quality cryo-EM reconstructions and comprehensive phylogenetic analysis represent clear strengths of the work. However, the functional conclusions can be strengthened by more rigorous validation and discussion. Addressing these points would strengthen the clarity and overall impact of the manuscript.

Reviewer #2

(Remarks to the Author)
The manuscript „A family of archaeal hibernation factors …" by Nissley et al. concentrates on the four sri genes/Sri proteins of Methanosarcina acetivorans. A quadruple gene deletion mutant (delta sriA – sriD) war generated and it was shown that it has a longer lag phase during outgrowth after inoculation of late stationary phase cells into fresh medium in comparison to the wildtype. In addition, the concentration of the 30S rRNA was much lower in the mutant than in the wildtype in late stationary phase, explaining the growth phenotype.
Based on that observation, the 70S ribosome was reconstituted together with SriA/SriB, SriD, and SriC/SriD, and high-resolution structures were generated. It was verified that the two pairs of proteins indeed bind to the ribosome, and the details of the binding interactions were elucidated. It was revealed that the binding sites overlap, and that thus SirAB and SriCD cannot bind simultaneously to the same ribosome.
Subsequently the phylogenetic distribution of the four Sri proteins as well as the proteins Hib and Dri were resolved, and it was found that Sri and Hib have a more or less mutual exclusive distribution.
All in all, the manuscript is very interesting and provides the novel information that the Sri proteins are ribosome hibernation factors that protect especially the 30S rRNA from degradation in late stationary phase. It also unravels that archaea contain at least two different ribosome hibernation factors, Sri and Hib.
Taken together, I am in favor of publication, although some important questions concerning the biological roles of the Sri proteins have not been addressed.
For example, it would have been very interesting to know the copy numbers of the Sri proteins and the ribosomes in exponential and late stationary phase. Are there enough Sri protein pairs in the cell to protect all ribosomes, or is only a small fraction protected? It was shown that the sir genes are expressed already during exponential growth. Are the transcripts translated, and if yes, how are the Sri proteins inhibited from ribosome binding during exponential growth and early stationary phase? The ribosomal subunits were isolated under ribosome dissociating conditions. It would have been very easy and interesting to isolate the ribosomes also under non-denaturating conditions to unravel whether 70S ribosomes persist during the transition into stationary phase in the wildtype, in contrast to the mutant. Etc.

I have a few minor comments that should be addressed prior to publication.
- Line 43. Why is the section entitlted "Main" instead of "Introduction"?
- L. 82. A "four gene cluster" cannot be composed of "one N-terminal Dri homolog and on C-terminal Dri homolog"
- L. 103. If the four sri genes were under the control of a single promoter, an equal read coverage would be expected from sriA to sriD, which is not the case. While it cannot be excluded that there is indeed a single promoter and the polycistronic transcript is processed between the ORFs, it is as likely possible that there is one promoter upstream of sriA and three additional promoters upstream of the other three sri genes. Many gene clusters in prokaryotes contains multiple promoters.
- L. 114. The results indicate that the MCR concentration was not "titrated", but one MCR limited state was compared to one MCR sufficient state.
- L. 120. When the "downregulation" "decreases", this means that the concentration in fact increases. I suspect that was not was the authors really meant.
- Fig. 1c/d. The transcript levels of the four sri transcripts are very different according to Fig. 1d (at least a fivefold difference between sriA and sriC/D), which is in sharp contrast to the read coverage shown in Fig. 1c.
- 184ff. It should be discussed how binding of SriA/SriB to the LARGE subunit of the ribosome can protect the SMALL subunit from degradation.
- L. 189ff. The density of a previously undescribed rProtein was detected. How did the authors come from the density (L. 189) to the protein sequence (L. 193)?
- L. 250. It should be discussed why SriC was only detected in a small portion of particles. It could be that the affinity of SriC to the ribosome is much lower than that of the other three Sri proteins (then why?), or that SriC is not a bona fide ribosome binding protein at all, but binding was enforced by the high concentrations during reconstitution.
- L. 289. Sri/hib/dri genes are not present in "most archaea", 65% is not "most". And sri genes are present in only 23%, and in most cases not all four genes.
- L 316. "different ribosomes" is misleading. It implies that ribosomes of different composition and/or different modifications exist (like in plants). I do not know any indications for that in archaea.

- L. 317ff. Why is only the SSU degraded and not the LSU? And why are proteins needed that bind to the LSU when the SSU is much more fragile? The circularization of the 23S rRNA is not really an explanation after the observed action of endonucleases that generate open 5'- and 3'-ends.
- L. 331. Genes are "expressed", proteins not.
- L. 340. Sri proteins are not really present in "multiple archaeal phyla", Fig. 5 shows that the gene cluster is mainly confined to the Halobacteriota and additionally occurs only in a few species in other phylogenetic groups, indicating lateral gene transfer. Also the presence of bicistronic gene clusters in a few scattered additional species indicates lateral gene transfer.
- L. 451. The elution buffer is stated to contain only "20 mM imidazole", like the wash buffer. This seems to be highly unlikely.
- L. 488. Isolation without dissociation would have been easy and interesting (see above).

Reviewer #3

(Remarks to the Author)
This manuscript looks at ribosome hibernation in archaea. There are many studies that have looked into ribosome hibernation in bacteria and eukaryotes, but very few in archaea, giving this study novelty and an advancement of understanding in the field.

The authors have discovered a family of ribosome hibernation factors in the methanogenic archaea M. acetivorans, consisting of four proteins designated SriA-SriD, whose genes sriA-sriD are found encoded together on an operon. They have looked at expression of the sri genes under different growth conditions, expressed and purified the four Sri proteins and shown that each Sri protein inhibits protein synthesis in vitro. Production of a knock-out strain of M. acetivorans that lacks all four sri genes showed that these cells take longer to recover after a prolonged stationary phase than the wild type strain. The sri knock-out strain also shows a reduction in its amount of small ribosomal subunit compared to the wildtype strain, suggestive of a protective role. Under stressful conditions, the sri knockout and wild type strains grew similarly. Sri proteins appear to be important in dormancy, but not stress conditions.

Purified Sri proteins were added to lysates of M. acetivorans, the resulting ribosome-Sri complexes purified and high resolution cryo-EM structures (2-3Å) determined. This has shown the binding sites for the Sri proteins, with one pair of Sri proteins binding to the large ribosomal subunit and the other pair binding to the small subunit. The authors have also looked into the phylogenetics of the genes across archaea and bacteria and seen that these hibernation factors are common across many archaeal phyla.

This is a well-written manuscript with concise text and clear figures, and the experimental work and subsequent analysis has been carried out robustly, using a number of different complementary techniques to look at the structure and function of this new family of hibernation factors.

There are a few minor points that I noted:

Line 90 – what do you mean by 'potential for their modular use in different physiological contexts and environments'?

Fig 1b – are these figures to scale eg SriB is longer than SriA? It might be easier to see if the four are aligned with each other on the left hand side.

Line 105 – is the dual CBS domain gene, MA4647, Dri?

Fig 1e and line 117 – what happens to sriB transcription in energy depleted cells? In addition I would have found a brief description in the text or the legend as to how to interpret this plot useful.

Line 859 – typo tetracycline

Supp. Fig 3 /line 70 – typo – post-transcriptional modifications

Supp. Fig 3 / line 196 - the text talks about Mg binding sites, but there is no image of these in Supp. Fig 3.

Supp. Fig 4 – typos in the titles of part a – middle and right – focused

Ext data Fig4d / line 967 - in blue and red, are these SriA and SriB models? Legend says they are the SriA and SriB binding sites which suggests to me the pocket that SriA and SriB fit in to. In the yellow part of this figure, is it possible to label some of the helices to correspond with part c?

Supp. Fig 5 / line 82. Typo - SriA instead of SirA.

Fig 3d / line 224– I found a little unclear and was unsure what you meant by the text in the results section. Is tRNA in this figure? Is there a better way to show this? Maybe by adding a second view?

Supp. Fig 5c – could you add N and C termini to SriB?

Fig 4b title – typo overlaid rather than overlayed

Extended data Fig 5a and 5b – I feel these would be clearer if H69 was in the same orientation in both a and b.

Ext data Fig5e – I didn’t see reference to this figure in the text

Line 427 – typo - remove first instance of plates

Line 681 - there is reference to supplementary file 10, but this was not included.

Additional supplementary figures – I would like to see a typical micrograph and 2D class averages for the structures. I would also like to see a figure of each hibernation factor overlaid on its cryo-EM map to show the fit to the density.

Additional question – did the authors try to determine structures of each Sri protein individually bound to ribosomes? If so, this would be worth mentioning in the manuscript. I am not asking this to be done for this manuscript, but would personally be interested to see if SriB, for example, bound in a different conformation when SriA is not present.

Version 1:

Reviewer comments:

Reviewer #1

(Remarks to the Author)
I have carefully evaluated the revised manuscript and I am very impressed by the quality of the revisions. The authors did an excellent job addressing the previous concerns, especially considering the short time frame in which the additional analyses and modifications were completed. The manuscript is now stronger both scientifically and conceptually. Importantly, the revised version reads as a cohesive story. Overall, I believe this study represents an important contribution to the field and is fully suitable for publication in Nature Communications. I therefore support acceptance of the manuscript.

Reviewer #2

(Remarks to the Author)
The authors have reacted adequately to most of the points raised by all three reviewers. At a few places they did not follow the suggestions of the reviewers, however, these few points are not important enough to inhibit publication. Therefore, in my opinion the revised manuscript can be published in the present form.

Reviewer #3

(Remarks to the Author)
The authors have addressed all my comments.

Open Access This Peer Review File is licensed under a Creative Commons Attribution 4.0 International License, which permits use, sharing, adaptation, distribution and reproduction in any medium or format, as long as you give appropriate credit to the original author(s) and the source, provide a link to the Creative Commons license, and indicate if changes were made.
In cases where reviewers are anonymous, credit should be given to 'Anonymous Referee' and the source.
The images or other third party material in this Peer Review File are included in the article's Creative Commons license, unless indicated otherwise in a credit line to the material. If material is not included in the article's Creative Commons license and your intended use is not permitted by statutory regulation or exceeds the permitted use, you will need to obtain permission directly from the copyright holder.
To view a copy of this license, visit https://creativecommons.org/licenses/by/4.0/

REVIEWER COMMENTS

Reviewer #1 (Remarks to the Author):

The manuscript by Nissley, Williams et al., entitled " A family of archaeal hibernation factors that bind in tandem and protect ribosomes in dormant cells", presents a comprehensive study of a set of ribosome hibernation factors (SriA–SriD) in the methanogenic archaeon Methanosarcina acetivorans. By combining transcriptomics, genetics, in vitro translation assays, cryo-EM, and phylogenetic analysis, the authors propose that Sri proteins repress translation, protect ribosomes during prolonged stationary phase, and bind ribosomes as paired factors at two distinct functional sites. The cryo-EM reconstructions are of excellent quality, and the phylogenetic analysis convincingly demonstrates that Sri homologs are broadly distributed across archaea. Overall, the work is strong and represents a very valuable contribution to the field of archaeal ribosome biology. However, several aspects of the functional analysis and mechanistic interpretation require clarification or additional experimental support.

Major comments:

1. The primary in vivo phenotype observed in the ΔsriABCD strain is an extended lag phase (28 days post-exponential growth). However, M. acetivorans is known to encode additional ribosome hibernation systems, including MRDF and potentially Hib homologs, which could compensate for the loss of Sri proteins. This potential functional redundancy complicates the interpretation of the phenotype. The authors should therefore discuss more explicitly how the presence of alternative hibernation factors might buffer the effects of the sri deletion.

We thank the reviewer for raising this important point. There are no Hib orthologs in *M. acetivorans,* but we agree that compensatory overexpression of MRDF could contribute to the increased lag time we observe in Δ*sriABCD.* While we cannot conclusively rule out a potential role for MRDF, we observe no significant difference in expression of MRDF genes, PurH (MA4012) and aEF2 (MA1257) between WT and Δ*sriABCD* at 28 days post-exponential phase (see table of DEseq values below), when transcription is already at very low levels. Additionally, in energy depleted cells, where *sriACD* are upregulated, PurH is downregulated compared to WT in the dataset shown in Fig. 1D-E. These data support a model where these sets of hibernation factors have distinct roles. We have expanded on this subject discussion to more clearly address this.

MRDF genes DEseq values, Δ*sriABCD*_28dpe vs WT_28dpe

Gene	log_2(fold change)	P value	Q value
PurH	0	1	1
aEF2	5.43638627	0.01549862	0.31533959

2. All functional conclusions rely solely on the quadruple ΔsriABCD knockout. Because SriA/B associate with the large ribosomal subunit, whereas SriC/D bind the small subunit, and because these factors display distinct in vitro activities, the use of only a combined deletion makes it difficult to assign specific roles to each module. If possible, generating and analyzing ΔsriAB and ΔsriCD strains would potentially allow separation of LSU- and SSU-specific effects and provide a more mechanistically precise interpretation.

We appreciate the reviewer's insightful suggestion. To this point, we generated three new tet-inducible complementation strains (Δ*sriABCD*::PmcrB(tetO1)-*sriABCD*; Δ*sriABCD::*PmcrB(tetO1)-*sriAB*, and Δ*sriABCD::*PmcrB(tetO1)-*sriCD*) and analyzed their growth phenotype. Please see (**Fig. 5, Supplementary Fig. 14, and Supplementary Table 9**) and the corresponding updated text.

In summary, we observe that overexpression of *sriABCD* in the Δ*sriABCD* deletion mutant rescues the wildtype (WT) lag phenotype, and indeed even reduces lag time relative to WT. The *sriAB* complementation strain phenocopies the *sriABCD* complementation strain. In contrast, overexpression of SriCD alone does phenocopy WT.

3. Translation inhibition is evaluated at only a single Sri concentration (6 µM), and no estimate of the intracellular abundance of Sri proteins is provided. Without concentration-dependent curves in the in vitro translation assays, it is difficult to judge whether the observed inhibition reflects physiologically relevant interactions. Performing concentration-dependent or titration experiments would greatly strengthen the interpretation and biological relevance of the in vitro translation results.

We thank the reviewer for this comment. In the *in vitro* translation experiment we aimed to demonstrate that Sri proteins are capable of inhibiting translation consistent with their ability to occlude ribosome active sites. To further probe the physiological impacts of Sri concentrations *in vivo*, we took advantage of our tetracycline inducible expression system and overexpressed SriABCD, AB, and CD, respectively, at three levels of induction in the WT background. In brief, at all levels of induction, we observe no difference in lag or growth rate when strains were outgrown from exponential phase starter cultures. Please see **Supplementary Fig. 5**. We additionally tested two levels of *sri* expression in the Δ*sriABCD* background complementation strains in **Fig. 5**, as described in the response to comment 2 above. Altogether this data suggests that while Sri proteins can inhibit translation *in vitro*, Sri interactions with the ribosome are highly regulated *in vivo*, even when Sri concentrations are increased. We have added these points to the discussion.

4. The cryo-EM data indicate that SriC occupancy is low, even when SriC and SriD are supplied at high concentrations. If SriC has intrinsically low affinity for the ribosome, this raises questions about its stability and functional relevance in vivo. The authors should therefore discuss whether SriC binding is subject to regulation, for example through post-translational modifications or nucleotide/cofactor dependence.

This is an interesting point. We note that since Sri proteins were recombinantly expressed and Sri-ribosome complexes were reconstituted, the distribution of Sri-ribosome states is not necessarily indicative of natural Sri loading in stationary phase. Additionally, in all three cryo-EM data sets ~95% of ribosome particles have E-site tRNA occupancy, which overlaps with the SriC binding site. SriC loading is likely low in these datasets because of the presence of E-site tRNA, and SriC may therefore mainly target ribosomes that are not bound to tRNA *in vivo*. We have also seen evidence in our data that Sri proteins are regulated *in vivo* as indicated in response to point 3 above, and this may play a role in the low SriC occupancy. We have added these points to the discussion.

5. The authors argue that circularization of the 23S rRNA protects the LSU from exonucleolytic degradation. However, they also report a 21% reduction in domain IV coverage in the ΔsriABCD strain due to endonucleolytic cleavage. The authors should clarify in the manuscript whether SriA/SriB binding overlaps with or shields this region and whether the degradation site lies near their binding site, to better reconcile LSU protection with the observed domain IV degradation.

We thank the reviewer for their comment. There is substantial overlap between the regions of domain IV with reduced RNA-seq coverage in stationary phase and the binding sites of SriA and SriB. We have moved this data to **Fig. 3f-g** to better highlight this point, and we have clarified the text

Minor comments:

1. The cryo-EM analysis identifies two coexisting ribosome populations: one bound by SriA/B and the other by SriD, each associated with a distinct small subunit rotational state. This is an intriguing observation, as it implies the presence of functionally distinct hibernated ribosome states. However, the manuscript does not explore the implications of this finding. The authors should discuss what conditions might determine the distribution between these states.

We thank the reviewer for their comment. We agree that the different hibernation states have interesting implications for regulation. In **Fig. 1**, we show that while sri genes are transcribed polycistronically, *sriA* and *sriB* mRNA levels are lower than those for *sriC* and *sriD*. Different levels of sri expression could be used to control the distribution of ribosomes bound to SriAB or SriCD, which could be a way to regulate ribosome hibernation. We also find that complementation of *sriAB* and *sriCD* modules alone results in varying degrees of rescue of the *sri* KO lag phase extension we observe *in vivo* **(Fig. 4, Supplementary Fig. 14, and Supplementary Table 9)**. We have expanded this topic in the discussion**.**

2. The proposed role of CBS domains in nucleotide sensing is also an important aspect. Given that CBS domains typically bind adenosine nucleotides and that sri expression is linked to cellular energy status, it would be useful to indicate whether the Sri CBS domains contain adenosine-binding motifs. Clarifying the structural features of these domains would strengthen the mechanistic discussion.

We agree Sri regulation by nucleotides is an interesting aspect. We have not observed direct evidence of this in our experiments. However, since this manuscript was submitted, another study of SriC and SriD orthologs (found in another methanogen and referred to as ARC) was posted on *bioRxiv* in which the authors found that AMP and ATP regulate ARC-ribosome complex formation (https://doi.org/10.64898/2026.01.19.700304). These complementary findings, and the presence of adenosine binding motifs in some Sri proteins, suggests that Sri proteins bind nucleotides, but this is speculative and better left for future study. We have expanded the discussion on this topic.

In summary, the study by Nissley, Williams et al. represents a significant contribution by providing a structural and evolutionary framework for archaeal ribosome hibernation. The high-quality cryo-EM reconstructions and comprehensive phylogenetic analysis represent clear strengths of the work. However, the functional conclusions can be strengthened by more rigorous validation and discussion. Addressing these points would strengthen the clarity and overall impact of the manuscript.

We thank the reviewer for their helpful comments.

Reviewer #2 (Remarks to the Author):

The manuscript „A family of archaeal hibernation factors …" by Nissley et al. concentrates on the four sri genes/Sri proteins of Methanosarcina acetivorans. A quadruple gene deletion mutant (delta sriA – sriD) war generated and it was shown that it has a longer lag phase during outgrowth after inoculation of late stationary phase cells into fresh medium in comparison to the wildtype. In addition, the concentration of the 30S rRNA was much lower in the mutant than in the wildtype in late stationary phase, explaining the growth phenotype.
Based on that observation, the 70S ribosome was reconstituted together with SriA/SriB, SriD, and SriC/SriD, and high-resolution structures were generated. It was verified that the two pairs of proteins indeed bind to the ribosome, and the details of the binding interactions were elucidated. It was revealed that the binding sites overlap, and that thus SirAB and SriCD cannot bind simultaneously to the same ribosome.
Subsequently the phylogenetic distribution of the four Sri proteins as well as the proteins Hib and Dri were resolved, and it was found that Sri and Hib have a more or less mutual exclusive distribution.
All in all, the manuscript is very interesting and provides the novel information that the Sri proteins are ribosome hibernation factors that protect especially the 30S rRNA from degradation in late stationary phase. It also unravels that archaea contain at least two different ribosome hibernation factors, Sri and Hib.
Taken together, I am in favor of publication, although some important questions concerning the biological roles of the Sri proteins have not been addressed.

We thank the reviewer for their interest in our study.

For example, it would have been very interesting to know the copy numbers of the Sri proteins and the ribosomes in exponential and late stationary phase. Are there enough Sri protein pairs in the cell to protect all ribosomes, or is only a small fraction protected?

As *M. acetivorans* is an emerging model system, we currently lack the technical advances required to assess protein copy number (i.e. there is currently no known benchmark protein in our system like, for example, actin in eukaryotic systems). While we agree this would be a fascinating question to address in future work, given the degree of technical advancement required, we respectfully posit that this experiment is out of the scope of this study.

It was shown that the sir genes are expressed already during exponential growth. Are the transcripts translated, and if yes, how are the Sri proteins inhibited from ribosome binding during exponential growth and early stationary phase?

We do not have direct evidence of *sriABCD* translation in our experiments. However, comprehensive work by Ammar Tufail et al. (https://doi.org/10.1038/s41467-024-53008-8) showed active translation of Sri proteins (MM_RS06765, MM_RS06770, MM_RS06775, and MM_RS06780), by ribosome profiling in actively growing *Methanosarcina mazei,* a close relative of *Methanosarcina acetivorans.* We have added this point to the discussion.

We emphatically agree that the regulation of Sri binding to the ribosome is an important question. One possibility is that Sri binding to the ribosome is regulated by the translation landscape and when translation is upregulated during growth, tRNAs and translation factors outcompete Sri proteins for ribosome binding. Because Sri proteins contain CBS domains, we also considered that nucleotide binding may play a role in their regulation, but we did not observe evidence of this in our experiments. However, since this manuscript was submitted, another study of SriC and SriD homologs (referred to as ARC) was posted on *bioRxiv* in which the authors found that AMP and ATP regulate ARC-ribosome complex formation (https://doi.org/10.64898/2026.01.19.700304).

The ribosomal subunits were isolated under ribosome dissociating conditions. It would have been very easy and interesting to isolate the ribosomes also under non-denaturating conditions to unravel whether 70S ribosomes persist during the transition into stationary phase in the wildtype, in contrast to the mutant. Etc.

We agree that it would be interesting to quantify 70S populations in the WT and mutant strains. However, we have found that *M. acetivorans* ribosomes dissociate during ultracentrifugation even under associating conditions (https://doi.org/10.1038/s41564-025-02065-w). Dissociation of archaeal ribosomes during ultracentrifugation has been observed in several species (https://doi.org/10.1093/nar/gkad661 and https://doi.org/10.1016/0300-9084(91)90179-5). We have included the data from WT lysate run on a sucrose gradient with 10 mM $MgCl_2$ in **Supplementary Fig. 3d** to clarify this point.

I have a few minor comments that should be addressed prior to publication.

- Line 43. Why is the section entitlted “Main” instead of “Introduction”?

We have changed the title to introduction

- L. 82. A “four gene cluster” cannot be composed of “one N-terminal Dri homolog and on C-terminal Dri homolog”

The four-gene cluster is composed of one N-terminal Dri homolog, one C-terminal Dri homolog, and two additional four tandem CBS domain-containing proteins.

- L. 103. If the four sri genes were under the control of a single promoter, an equal read coverage would be expected from sriA to sriD, which is not the case. While it cannot be excluded that there is indeed a single promoter and the polycistronic transcript is processed between the ORFs, it is as likely possible that there is one promoter upstream of sriA and three additional promoters upstream of the other three sri genes. Many gene clusters in prokaryotes contains multiple promoters.

Thank you for pointing out this important distinction. We have taken this point and updated the text to reflect that we can only conclude that *sriABCD* are transcribed polycistronically from the intergenic read coverage we observe.

- L. 114. The results indicate that the MCR concentration was not “titrated”, but one MCR limited state was compared to one MCR sufficient state.

The titration data is in **Supplementary Fig. 1**.

- L. 120. When the “downregulation” “decreases”, this means that the concentration in fact increases. I suspect that was not was the authors really meant.

In the titration data, rProtein mRNA levels increase as as MCR levels increase. We have changed the wording to avoid the double negative.

- Fig. 1c/d. The transcript levels of the four sri transcripts are very different according to Fig. 1d (at least a fivefold difference between sriA and sriC/D), which is in sharp contrast to the read coverage shown in Fig. 1c.

The data in **Fig. 1c** and **Fig. 1d-e** are from different datasets, respectively. We have updated the figure legend and **Fig. 1d** title to more clearly communicate this.

- 184ff. It should be discussed how binding of SriA/SriB to the LARGE subunit of the ribosome can protect the SMALL subunit from degradation.

We thank the review for this comment. SriA/SriB binding to the LSU in hibernating ribosomes can protect the SSU by stabilizing 70S complexes, which are more stable to exonucleolytic

degradation than isolated 30S subunits (https://doi.org/10.1038/s41586-024-07027-6). We have added this point to the discussion.

- L. 189ff. The density of a previously undescribed rProtein was detected. How did the authors come from the density (L. 189) to the protein sequence (L. 193)?

We identified this protein sequence using the automatic modeling software ModelAngelo. This has been updated in the main text.

- L. 250. It should be discussed why SriC was only detected in a small portion of particles. It could be that the affinity of SriC to the ribosome is much lower than that of the other three Sri proteins (then why?), or that SriC is not a bona fide ribosome binding protein at all, but binding was enforced by the high concentrations during reconstitution.

Please see our response to reviewer #1 point 4.

- L. 289. Sri/hib/dri genes are not present in "most archaea", 65% is not "most". And sri genes are present in only 23%, and in most cases not all four genes.

We have changed "most" to "the majority of".

- L 316. "different ribosomes" is misleading. It implies that ribosomes of different composition and/or different modifications exist (like in plants). I do not know any indications for that in archaea.

We have changed "different" to "separate" to try not to imply that the ribosomes are different in composition.

- L. 317ff. Why is only the SSU degraded and not the LSU? And why are proteins needed that bind to the LSU when the SSU is much more fragile? The circularization of the 23S rRNA is not really an explanation after the observed action of endonucleases that generate open 5'- and 3'-ends.

Small subunit degradation is a common stress response that has been observed in eukaryotes (https://doi.org/10.1016/j.molcel.2025.01.013) and bacteria (doi/10.1261/rna.2448911). It is thought that the SSU is degraded to a greater extent than the LSU, because it is the first subunit involved in translation initiation, which is downregulated during stress and dormancy.

SriA and SriB likely play both roles in stabilizing the SSU through the formation of 70S ribosomes and by protecting domain IV of the LSU from degradation (shown in **Fig. 3f-g**).

Circularization of the 23S rRNA may provide the LSU with protection from exonucleases before they are subjected to endonucleolytic cleavage in domain IV. This could help to decrease the amount of LSU degradation compared to the SSU. While we found depletion of domain IV reads

in the 23S rRNA, we also observe a population of full length 23S rRNA from extended stationary phase (shown in **Fig. 2g**), suggesting that some 23S rRNAs are not subjected to endonucleolytic cleavage.

- L. 331. Genes are “expressed”, proteins not.

We have changed this to say “sri genes are expressed”.

- L. 340. Sri proteins are not really present in “multiple archaeal phyla”, Fig. 5 shows that the gene cluster is mainly confined to the Halobacteriota and additionally occurs only in a few species in other phylogenetic groups, indicating lateral gene transfer. Also the presence of bicistronic gene clusters in a few scattered additional species indicates lateral gene transfer.

We thank the reviewer for their comment. While the full sri gene cluster is mainly confined to Halobacteriota, it is not exclusively found in Halobacteriota and we also find that sri genes are found in multiple other archaeal phyla, even though they are found in different combinations. Sri genes are prevalent in some smaller phyla which are visually less prominent in **Fig. 6** (previously fig. 5), such as Altiarchaeota, where 32/35 species have sri genes, and Hydrothermarchaeota, where 23/27 species have sri genes, while being scarce in several of the largest phyla, like Thermoplasmatota and Nanobdellota.

- L. 451. The elution buffer is stated to contain only “20 mM imidazole”, like the wash buffer. This seems to be highly unlikely.

We thank the reviewer for catching this mistake. The method section now reads 500 mM imidazole.

- L. 488. Isolation without dissociation would have been easy and interesting (see above).

We have addressed this point above.

Reviewer #3 (Remarks to the Author):

This manuscript looks at ribosome hibernation in archaea. There are many studies that have looked into ribosome hibernation in bacteria and eukaryotes, but very few in archaea, giving this study novelty and an advancement of understanding in the field.

The authors have discovered a family of ribosome hibernation factors in the methanogenic archaea M. acetivorans, consisting of four proteins designated SriA-SriD, whose genes sriA-sriD are found encoded together on an operon. They have looked at expression of the sri genes under different growth conditions, expressed and purified the four Sri proteins and shown that each Sri protein inhibits protein synthesis in vitro. Production of a knock-out strain of M. acetivorans that lacks all four sri genes showed that these cells take longer to recover after a prolonged stationary phase than the wild type strain. The sri knock-out strain also shows a

reduction in its amount of small ribosomal subunit compared to the wildtype strain, suggestive of a protective role. Under stressful conditions, the sri knockout and wild type strains grew similarly. Sri proteins appear to be important in dormancy, but not stress conditions.

Purified Sri proteins were added to lysates of M. acetivorans, the resulting ribosome-Sri complexes purified and high resolution cryo-EM structures (2-3Å) determined. This has shown the binding sites for the Sri proteins, with one pair of Sri proteins binding to the large ribosomal subunit and the other pair binding to the small subunit. The authors have also looked into the phylogenetics of the genes across archaea and bacteria and seen that these hibernation factors are common across many archaeal phyla.

This is a well-written manuscript with concise text and clear figures, and the experimental work and subsequent analysis has been carried out robustly, using a number of different complementary techniques to look at the structure and function of this new family of hibernation factors.

We thank the reviewer for their comments.

There are a few minor points that I noted:

Line 90 – what do you mean by ‘potential for their modular use in different physiological contexts and environments’?

We have changed this statement to ‘potential for their use in different physiological contexts and environments’ to better highlight that CBS domain-containing hibernation factors are likely used by archaea in a variety of stress conditions across many environments.

Fig 1b – are these figures to scale eg SriB is longer than SriA? It might be easier to see if the four are aligned with each other on the left hand side.

Fig. 1b is to scale. SriB is longer because of the N-terminal extension that engages the LSU. We have added a scale bar to clarify this point.

Line 105 – is the dual CBS domain gene, MA4647, Dri?

The dual CBS domain gene is not Dri, but a gene of unknown function. Dri has 8 CBS domains in total.

Fig 1e and line 117 – what happens to sriB transcription in energy depleted cells? In addition I would have found a brief description in the text or the legend as to how to interpret this plot useful.

SriB expression does not change in energy depleted cells relative to WT. We have provided the WT FPKM values (**Fig. 1d**) of the highlighted genes in **Fig. 1e**, and changed the title of **Fig. 1d**

and updated the legend to make this more clear. We additionally added more context for the experiment referenced to improve interpretability **[lines 117-118]**. The volcano plot displays differential expression data wherein change in transcript level relative to some condition (WT) is plotted on the x axis as a function of significance of that change on the Y.

Line 859 – typo tetracycline

We have fixed this typo, thank you.

Supp. Fig 3 /line 70 – typo – post-transcriptional modifications

We have fixed this typo, thank you.

Supp. Fig 3 / line 196 - the text talks about Mg binding sites, but there is no image of these in Supp. Fig 3.

We have changed the text to only mention Zn binding sites.

Supp. Fig 4 – typos in the titles of part a – middle and right – focused

We have fixed these typos, thank you.

Ext data Fig4d / line 967 - in blue and red, are these SriA and SriB models? Legend says they are the SriA and SriB binding sites which suggests to me the pocket that SriA and SriB fit in to. In the yellow part of this figure, is it possible to label some of the helices to correspond with part c?

We thank the reviewer for their suggestions. We have modified the figure legend and labeled some of the rRNA helices.

Supp. Fig 5 / line 82. Typo - SriA instead of SirA.

We have fixed this typo, thank you.

Fig 3d / line 224– I found a little unclear and was unsure what you meant by the text in the results section. Is tRNA in this figure? Is there a better way to show this? Maybe by adding a second view?

We apologize for the confusion. tRNA is not shown in **Fig. 3d**. We are comparing the PTC conformation when tRNA is bound to the ribosome (grey, only PTC nucleotides are shown and not the tRNA) to the PTC confirmation when SriB is bound to the ribosome (tan). We have changed the figure caption to clarify this point.

Supp. Fig 5c – could you add N and C termini to SriB?

We have added N and C termini to the hibernation factors.

Fig 4b title – typo overlaid rather than overlayed

We have fixed this typo, thank you.

Extended data Fig 5a and 5b – I feel these would be clearer if H69 was in the same orientation in both a and b.

We thank the reviewer for their suggestion. Extended data Fig 5a and 5b (now **Supplementary Fig. 12)** were generated by aligning the LSU. In panel a, the apical portion of H69 (on the right) is disordered, which gives the appearance of H69 being in a different orientation than in panel b but the base of the helix is in the same orientation.

Ext data Fig5e – I didn't see reference to this figure in the text

We now reference this figure panel in the main text.

Line 427 – typo - remove first instance of plates

Corrected.

Line 681 - there is reference to supplementary file 10, but this was not included.

We apologize for the omission. It is included in the resubmission.

Additional supplementary figures – I would like to see a typical micrograph and 2D class averages for the structures. I would also like to see a figure of each hibernation factor overlaid on its cryo-EM map to show the fit to the density.

We have added micrographs and 2D class averages for each of the datasets in **Supplementary Fig. 19**. We have also added a figure showing the cryo-EM density for each of the Sri proteins in **Supplementary Fig. 9**.

Additional question – did the authors try to determine structures of each Sri protein individually bound to ribosomes? If so, this would be worth mentioning in the manuscript. I am not asking this to be done for this manuscript, but would personally be interested to see if SriB, for example, bound in a different conformation when SriA is not present.

We did not try to determine structures of each Sri protein individually. In our data, the only Sri protein that we found bound to the ribosome individually was SriD. It is worth noting that we were unable to find any ribosomes bound to SriA or SriB individually using 3D classification and were only able to find particles where both proteins were bound simultaneously.
