# Supplementary material for: A family of archaeal hibernation factors that bind in tandem and protect ribosomes in dormant cells
Source: Nat Commun. 2026 Jun 25;17:8083. doi: 10.1038/s41467-026-74727-0 (PMC13454478; doi:10.1038/s41467-026-74727-0)
Supplement: Supplementary file 3 [file 41467_2026_74727_MOESM1_ESM.pdf]

<u>Supplementary Information For:</u>

A family of archaeal hibernation factors that bind in tandem and protect ribosomes in dormant cells

Amos J. Nissley[1+], Madison C. Williams[2+], Yekaterina Shulgina[2-4], Roan W. Kivimae[1], Dipti D. Nayak[2,5*], and Jamie H. D. Cate[1-4,6*]

[1] Department of Chemistry, University of California, Berkeley, California, United States
[2] Department of Molecular and Cell Biology, University of California, Berkeley, California, United States
[3] Innovative Genomics Institute, University of California, Berkeley, California, United States
[4] California Institute for Quantitative Biosciences, University of California, Berkeley, California, United States
[5] Department of Plant and Microbial Biology, University of California, Berkeley, California, United States
[6] Molecular Biophysics and Integrated Bioimaging Division, Lawrence Berkeley National Laboratory, Berkeley, California, United States

+These authors contributed equally

*Correspondence should be addressed to Dipti Nayak (dnayak@berkeley.edu) or Jamie Cate (j-h-doudna-cate@berkeley.edu)

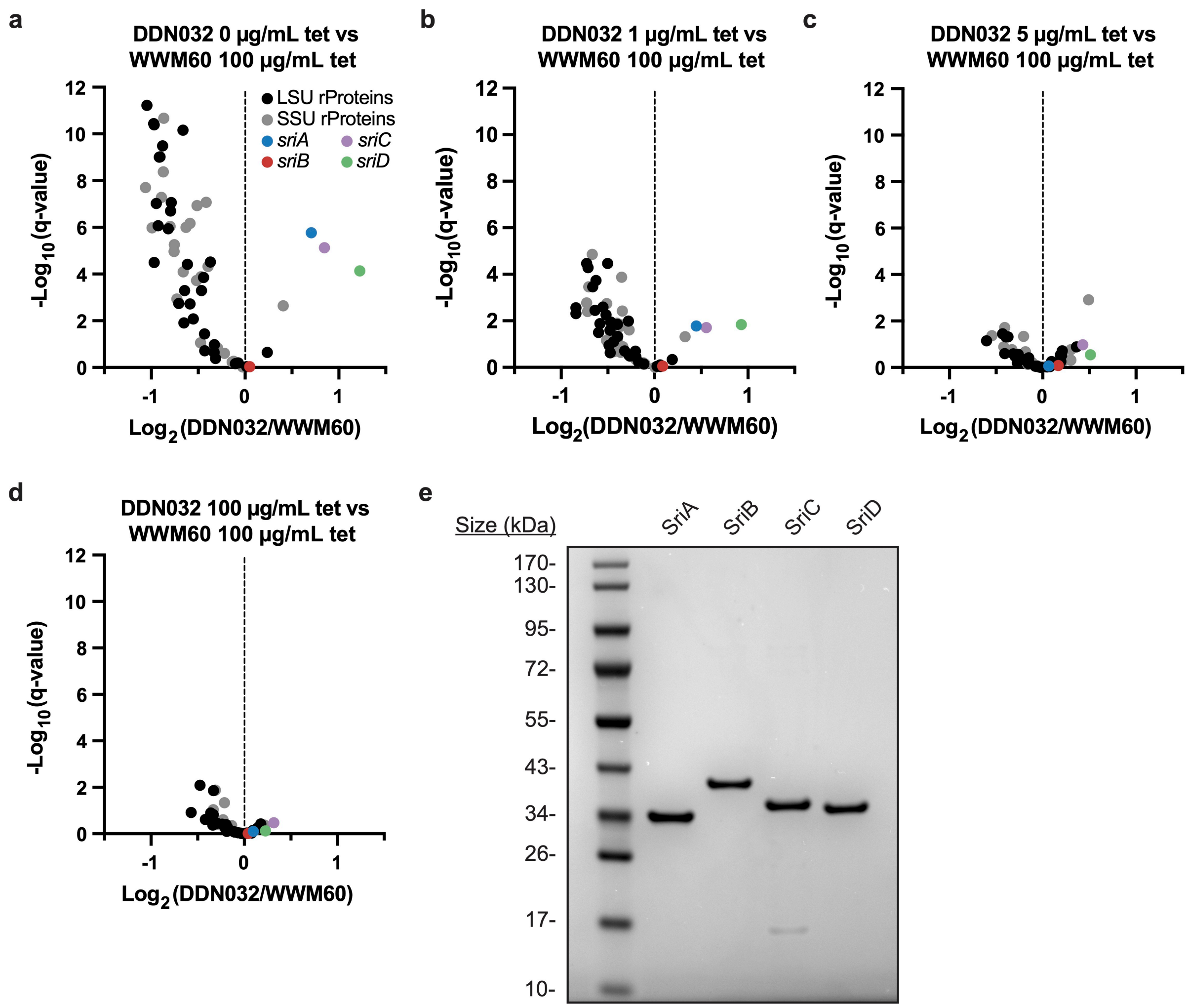

Supplementary Fig. 1: Differential expression of *sri* transcripts under Methyl-Coenzyme M Reductase (MCR) limitation and Sri protein purification. (a-d) Differential mRNA expression (significance and fold change) in DDN032 (tetracycline inducible MCR expression) and WWM60 (WT) *M. acetivorans* cultures. MCR expression was varied across panels a-d with increasing concentrations of tetracycline (tet). Panel a is reproduced from fig. 1 for comparison. (e) Recombinantly expressed and purified Sri proteins resolved on a 4-12% polyacrylamide gel stained with Coomassie brilliant blue.

a

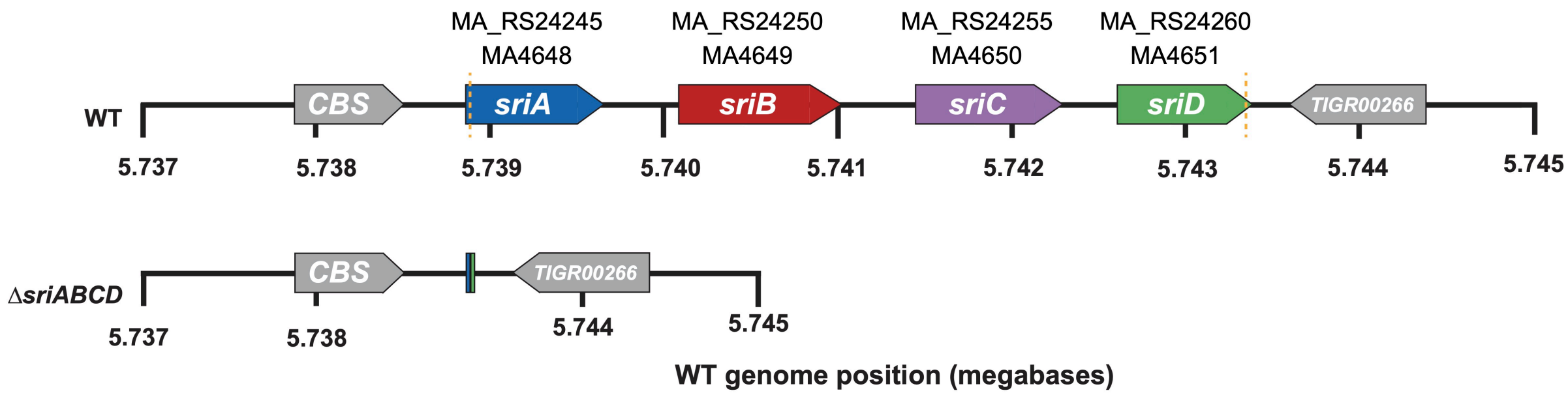

b

Predicted mutation

evidence	position	mutation	annotation	gene	description
MC JC	5,738,865	Δ4,501 bp		*[MA_RS24245]–[MA_RS24260]*	*[MA_RS24245], MA_RS24250, MA_RS24255, [MA_RS24260]*

Unassigned new junction evidence

		seq id	position	reads (cov)	reads (cov)	score	skew	freq	annotation	gene	product
*	?	WWM60	= 4367422	NA (NA)	66 (0.560)	27/296	1.4	78.6%	intergenic (+54/-552)	*MA_RS18440/MA_RS18445*	IS66-like element ISMac8 family transposase/methyltransferase domain-containing protein
–	?	WWM60	= 5412071	18 (0.150)					intergenic (-1216/-2)	*MA_RS22920/MA_RS22925*	winged helix-turn-helix domain-containing protein/cobalamin-dependent protein
*	?	WWM60	= 4734937	NA (NA)	55 (0.470)	23/296	2.0	84.6%	intergenic (-91/-307)	*MA_RS20090/MA_RS20100*	IS66-like element ISMac8 family transposase/phenylacetate--CoA ligase family protein
–	?	WWM60	5412064 =	10 (0.080)					intergenic (-1209/-9)	*MA_RS22920/MA_RS22925*	winged helix-turn-helix domain-containing protein/cobalamin-dependent protein
*	?	WWM60	= 5411299	84 (0.710)	39 (0.330)	23/296	2.0	40.6%	intergenic (-444/-774)	*MA_RS22920/MA_RS22925*	winged helix-turn-helix domain-containing protein/cobalamin-dependent protein
–	?	WWM60	= 5412006	30 (0.250)					intergenic (-1151/-67)	*MA_RS22920/MA_RS22925*	winged helix-turn-helix domain-containing protein/cobalamin-dependent protein

Supplementary Fig. 2. Δ*sriABCD* construction and whole genome sequencing analysis of the mutant strain (DDN482). (a) Schematic of the *sri* locus in WT (top) and CRISPR-edited knockout mutant, Δ*sriABCD* (bottom); dashed orange lines indicate boundaries of KO region. (b) Screenshot of Breseq[1] mutation prediction results from Δ*sriABCD* whole genome sequencing reads aligned to the WT genome with MA_RS annotations. MA4648 (*sriA*) is MA_RS24245; MA4649 (*sriB*) is MA_RS24250; MA4650 (*sriC*) is MA_RS24255; MA46451 (*sriD*) is MA_RS24260.

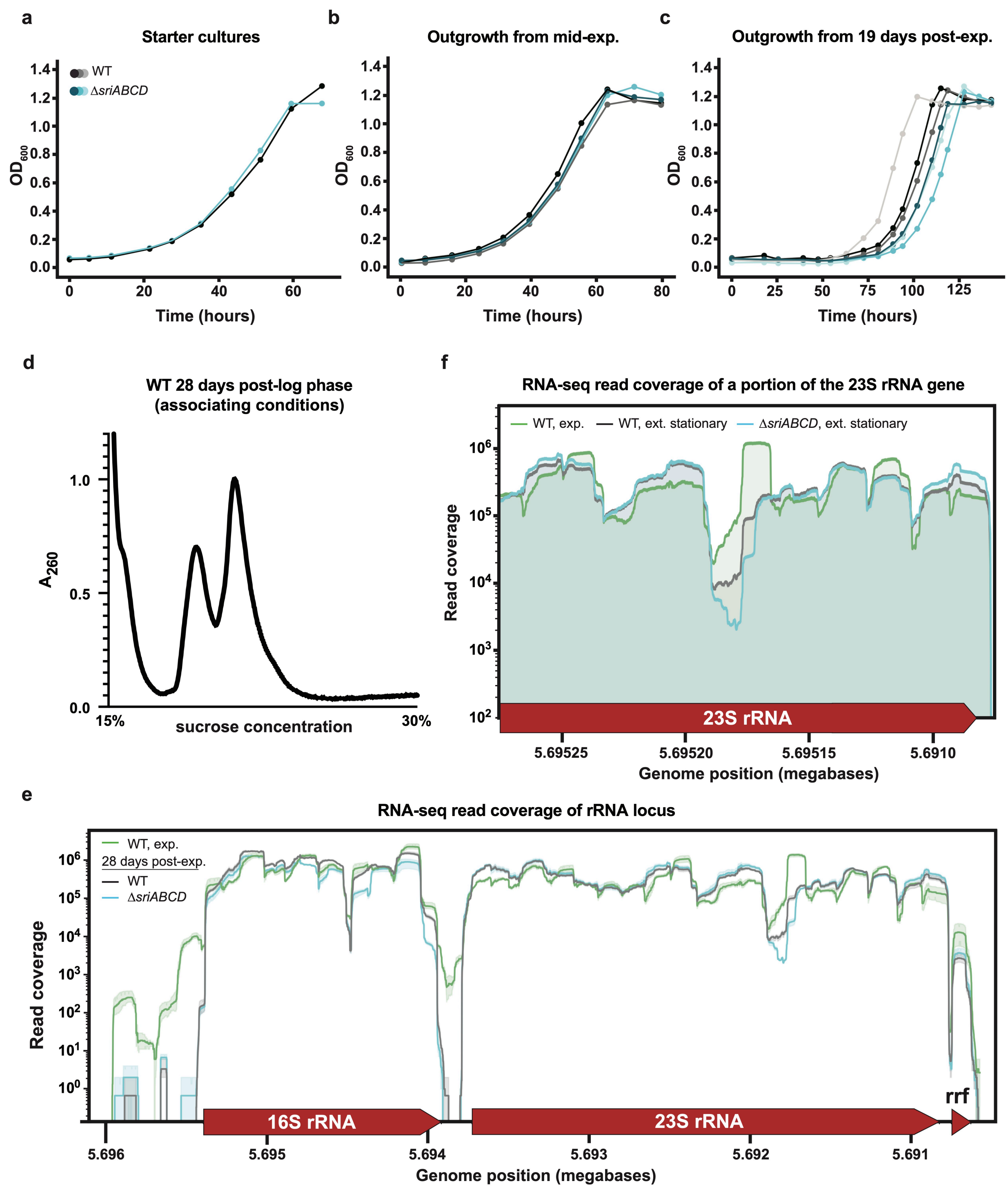

Supplementary Fig. 3: Supporting data for Fig. 2. (a-c) Additional stationary phase recovery growth curves; starter cultures, mid-exponential (mid-exp.) and 19 days post-exponential (post-exp.) outgrowth curves. Grey: WT - WWM60, parent strain; blue: Δ*sriABCD* - DDN482, harbors editing plasmid (pMW005). Mid-exp outgrowths cultured

in duplicate, all other outgrowths cultured in triplicate. (d) 0.75 mg of protein content from WWM60 (WT) in extended stationary phase was resolved on an associating 15-30% sucrose gradient. (e) Total RNA read coverage of an rRNA locus. The mean of 3 technical replicates per condition (strains and growth phases) is shown as a solid line and shading indicates the 95% confidence interval. (f) RNA-seq read coverage for a portion of the 23S rRNA gene. One representative replicate per strain is shown for visual clarity.

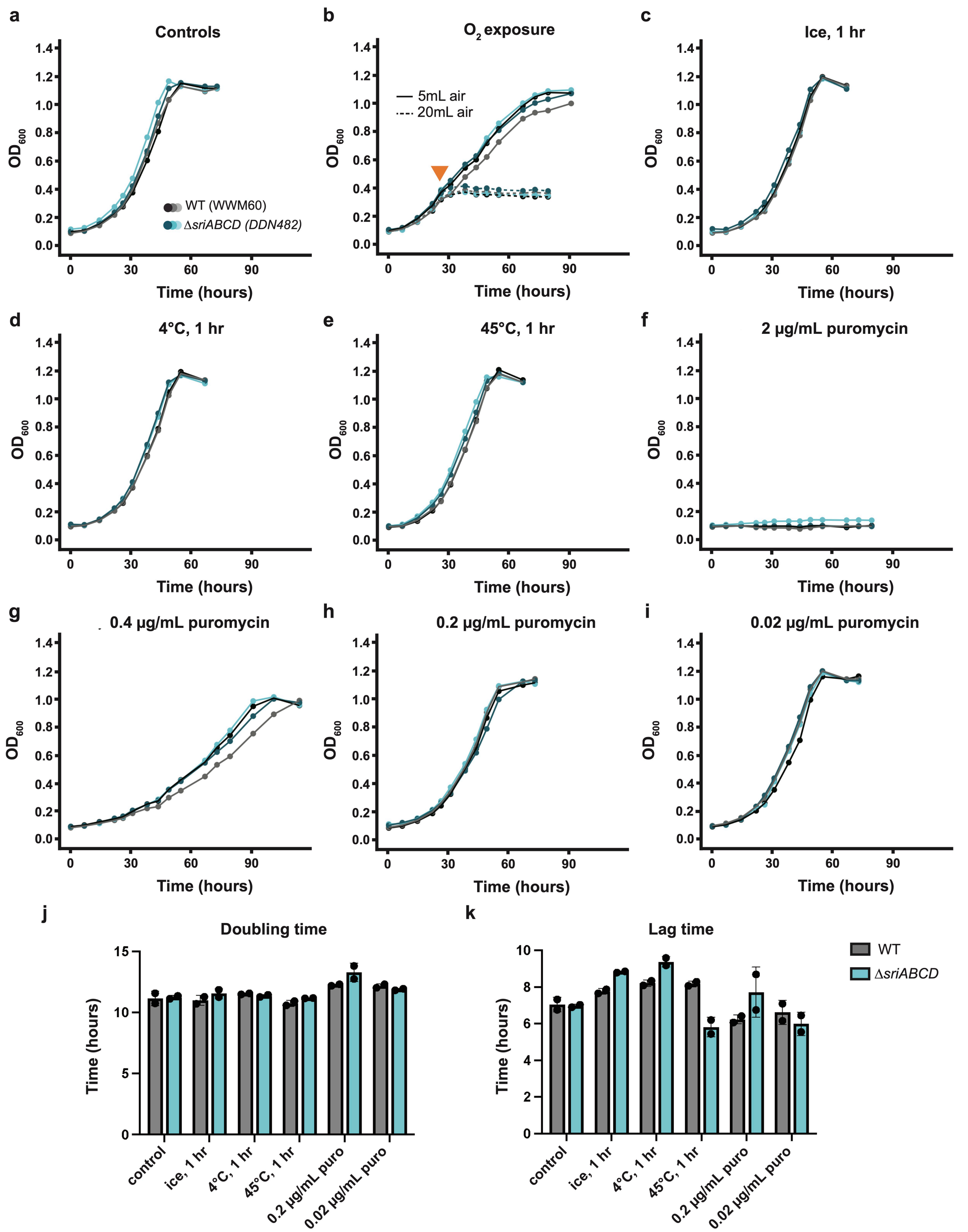

Supplementary Fig. 4: Challenging Δ*sriABCD* with various stressors yields no growth defect relative to WWM60 (WT). (a-i) WT (WWM60, grey) vs Δ*sriABCD* (blue) growth

curves under the following conditions. (a) Controls; standard conditions: 37 °C, anaerobic, 0 μg/mL puromycin. (b) 5 or 20 mL ambient, 0.22 μm filtered air injected into tubes in early exponential phase (indicated by orange carat). (c-e) 1hr incubation immediately after inoculation in ice, 4 °C and 45 °C, respectively; incubated at 37 °C for the remainder of the experiments. (f-i) Growth medium supplemented with 2, 0.4, 0.2, and 0.02 μg/mL puromycin (puro). (j) Doubling and (k) lag times calculated for cultures which grew exponentially (all except b, f, g). Error bars represent standard deviation. All conditions tested in duplicate. All Δ*sriABCD* mutant lag and doubling values were tested for statistical significance against respective WT controls with unpaired Welch's t-tests; no statistically significant differences were identified.

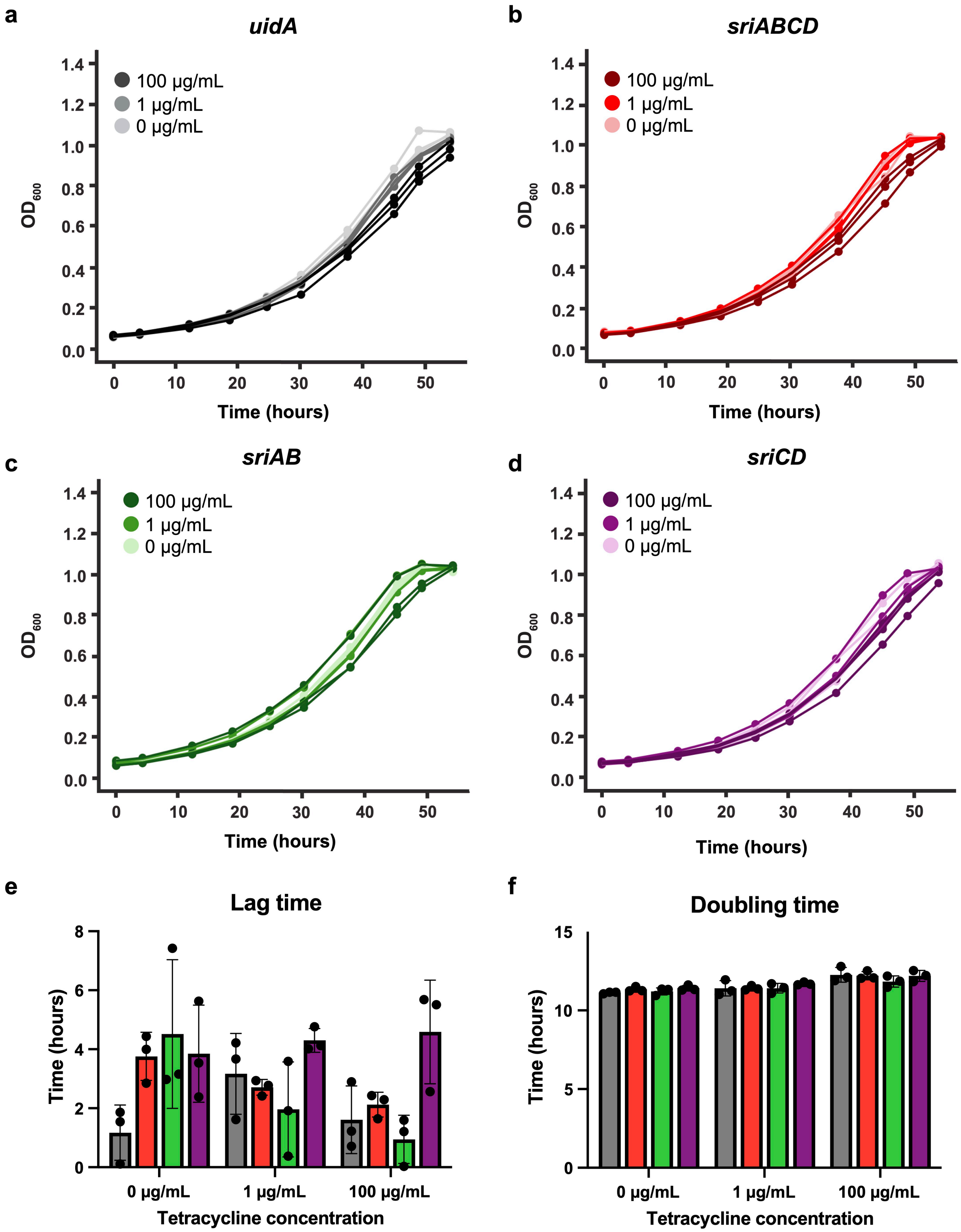

Supplementary Fig. 5: Overexpression of *sri* genes in exponential phase of WT cultures. (a-d) Growth curves of *M. acetivorans* strains harboring tetracycline (tet)

inducible expression constructs driving expression of (a) *uidA* (unrelated gene, control), (b) *sriABCD,* (c) *sriAB* (d) *sriCD.* Bar plots of (e) lag time and (f) doubling time for each strain and tet induction level. Tet was supplemented in the outgrowth media at the concentrations indicated (0μg/mL, 1μg/mL, or 100μg/mL); tet was not supplemented in starter cultures. Error bars represent standard deviation. All conditions were tested in triplicate. All lag and doubling values were tested for statistical significance against *uidA* controls with multiple unpaired Welch's t-tests; no statistically different values were found.

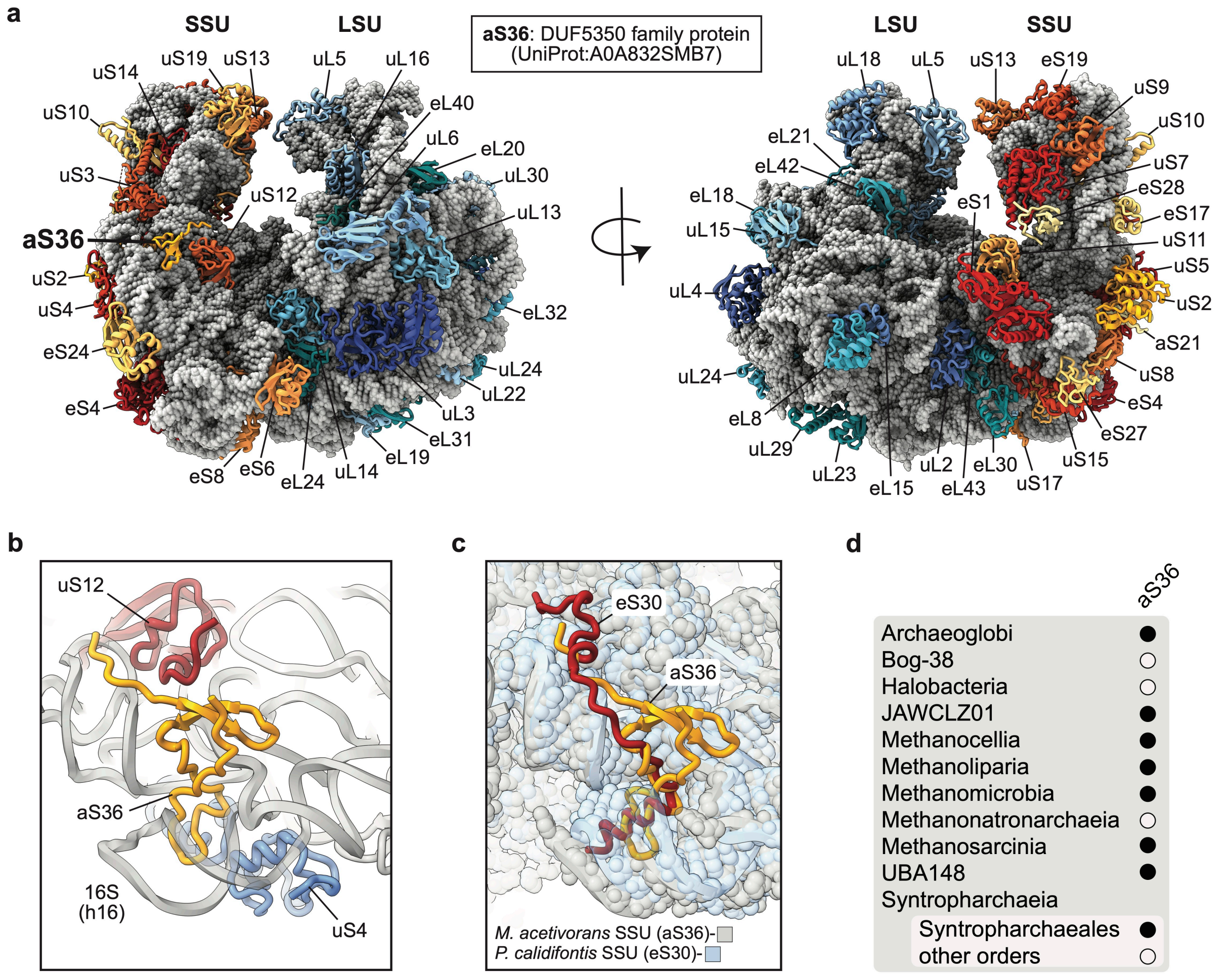

Supplementary Fig. 6: rProteins in the *M. acetivorans* ribosome. (a) rProteins are labeled on the model of the *M. acetivorans* 70S ribosome. The aS36 label is bolded and its previous annotation, including the accession code, is shown in the middle box. (b) aS36 is in proximity to uS4 and uS12 and interacts with 16S rRNA helix h16. (c) Overlay of the SSUs from *M. acetivorans* (grey) and *P. calidifontis* (blue, PDB:9E71[2]) showing the locations of aS36 and eS30, respectively. (d) Distribution of aS36 across the archaeal phylum Halobacteriota. Closed and open circles represent clades where aS36 homologs were or were not identified, respectively.

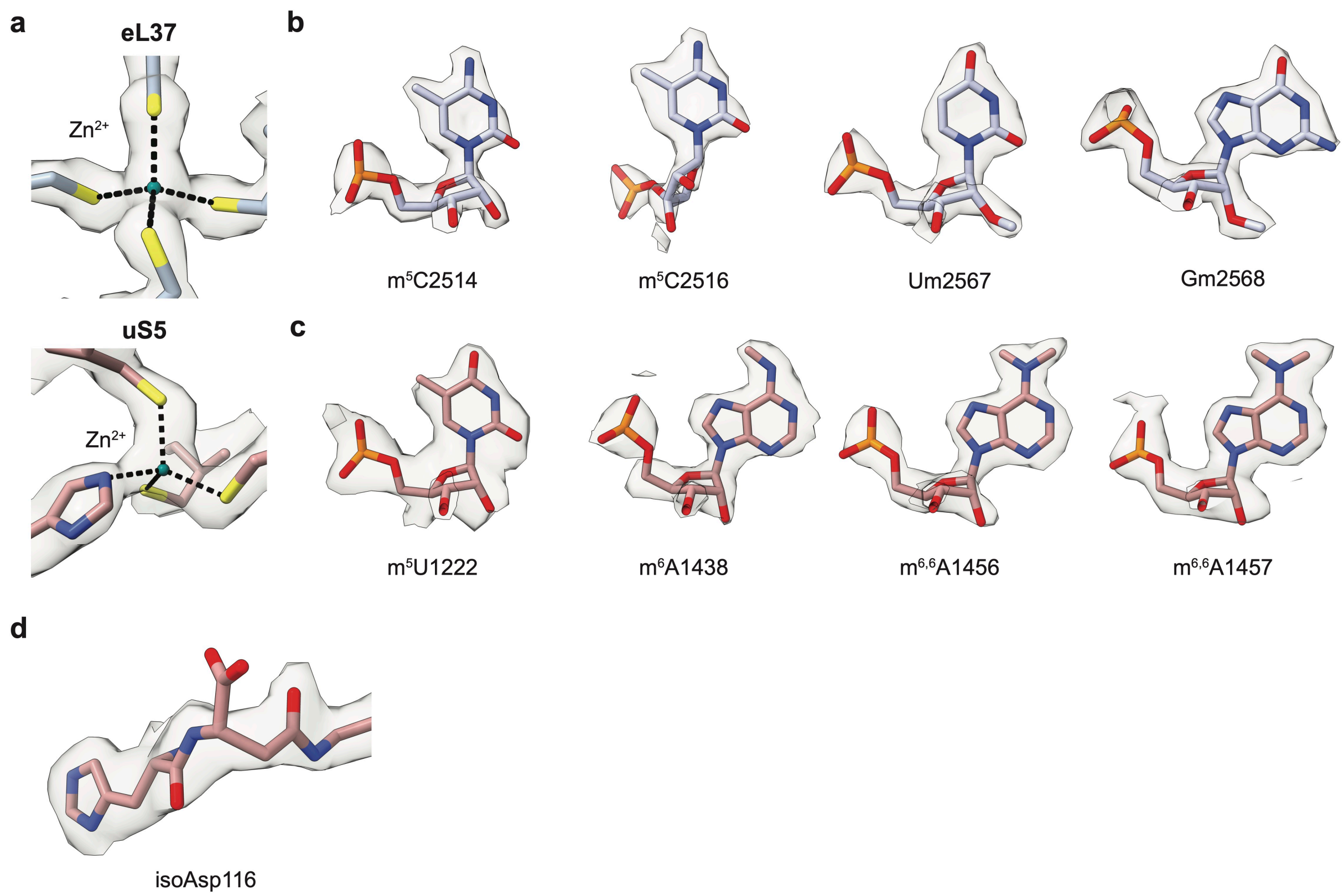

Supplementary Fig. 7: Metal binding sites and post-transcriptional and post-translational modifications in the *M. acetivorans* ribosome. (a) Zinc binding sites in LSU rProtein eL37 and SSU rProtein uS5 with cryo-EM density. (b-c) Cryo-EM density for post-transcriptional modifications in the LSU (b) and SSU (c). (d) Cryo-EM density for isoaspartate residue 116 in uS11.

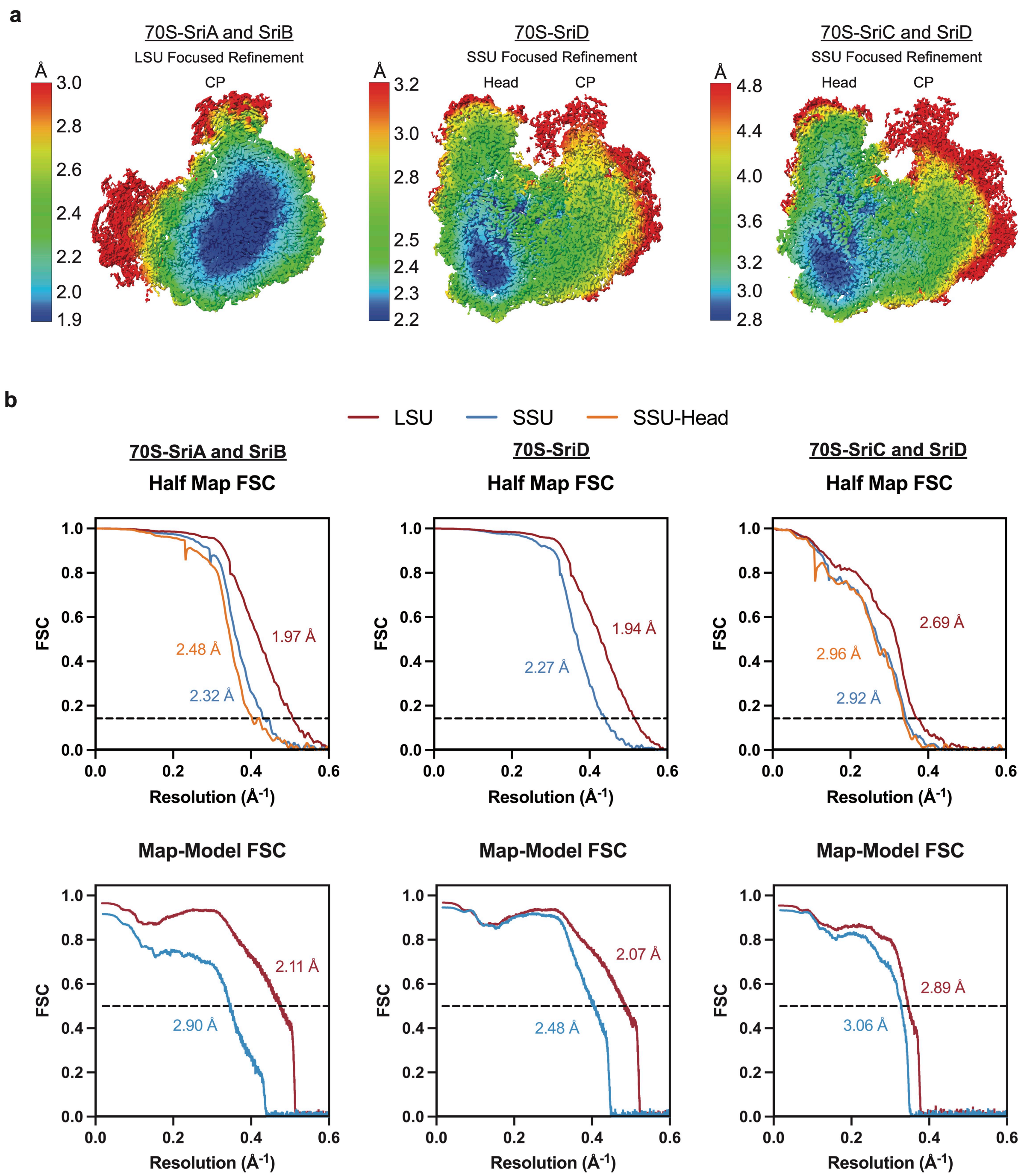

Supplementary Fig. 8: Resolution of cryo-EM maps. (a) Local resolution plotted on the LSU (left) or SSU (middle and right) focused refinement maps. The LSU central protuberance (CP) is labelled. (b) Half map (top) and map to model (bottom) FSCs for cryo-EM maps and models in this study.

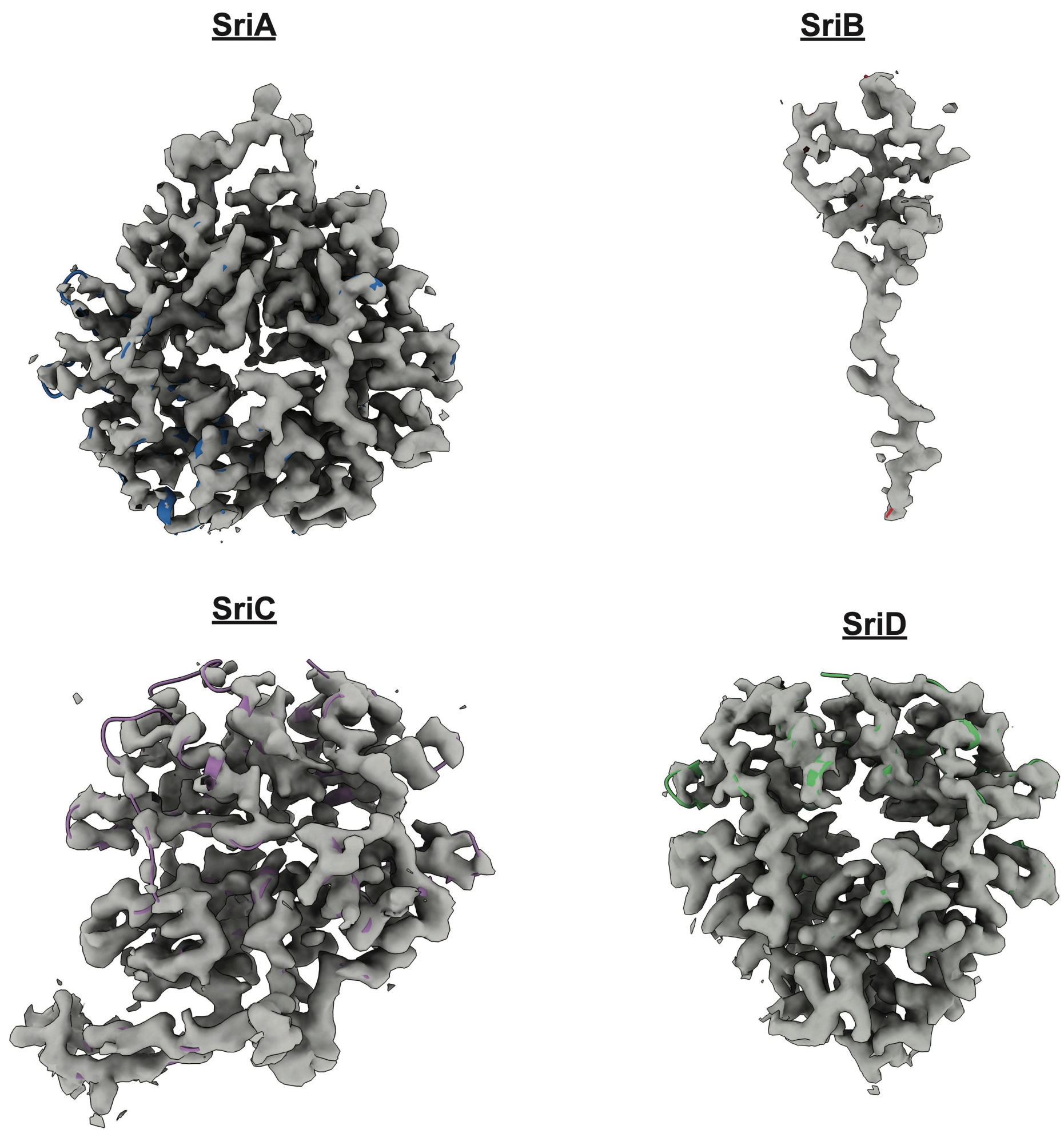

Supplementary Fig. 9: Cryo-EM density for SriA, SriB, SriC, and SriD overlaid on the molecular models.

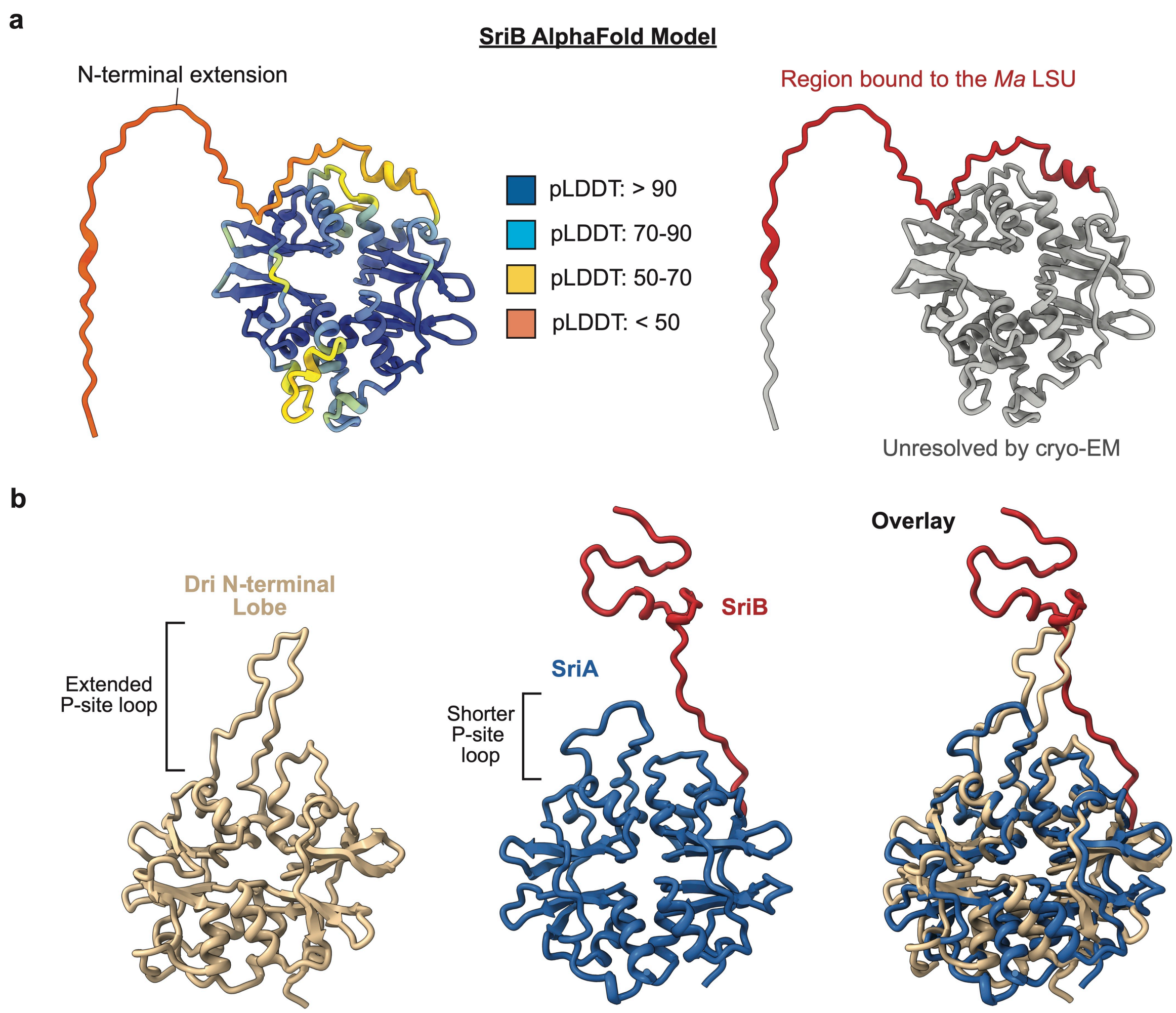

Supplementary Fig. 10: Structural features of SriA and SriB. (a) AlphaFold[3] prediction of SriB colored by (left) confidence score or (right) regions that are ordered (red) or disordered (grey) in the cryo-EM structure of SriB bound to the *M. acetivorans* ribosome. (b) Comparison of the Dri N-terminal lobe (left) with the SriA-SriB complex (middle). (right) Alignment of the *M. acetivorans* LSU in complex with SriA and SriB to the *P. calidifontis* LSU in complex with Dri (PDB:9E6Q[2]), highlighting the overlap in Sri and Dri binding sites.

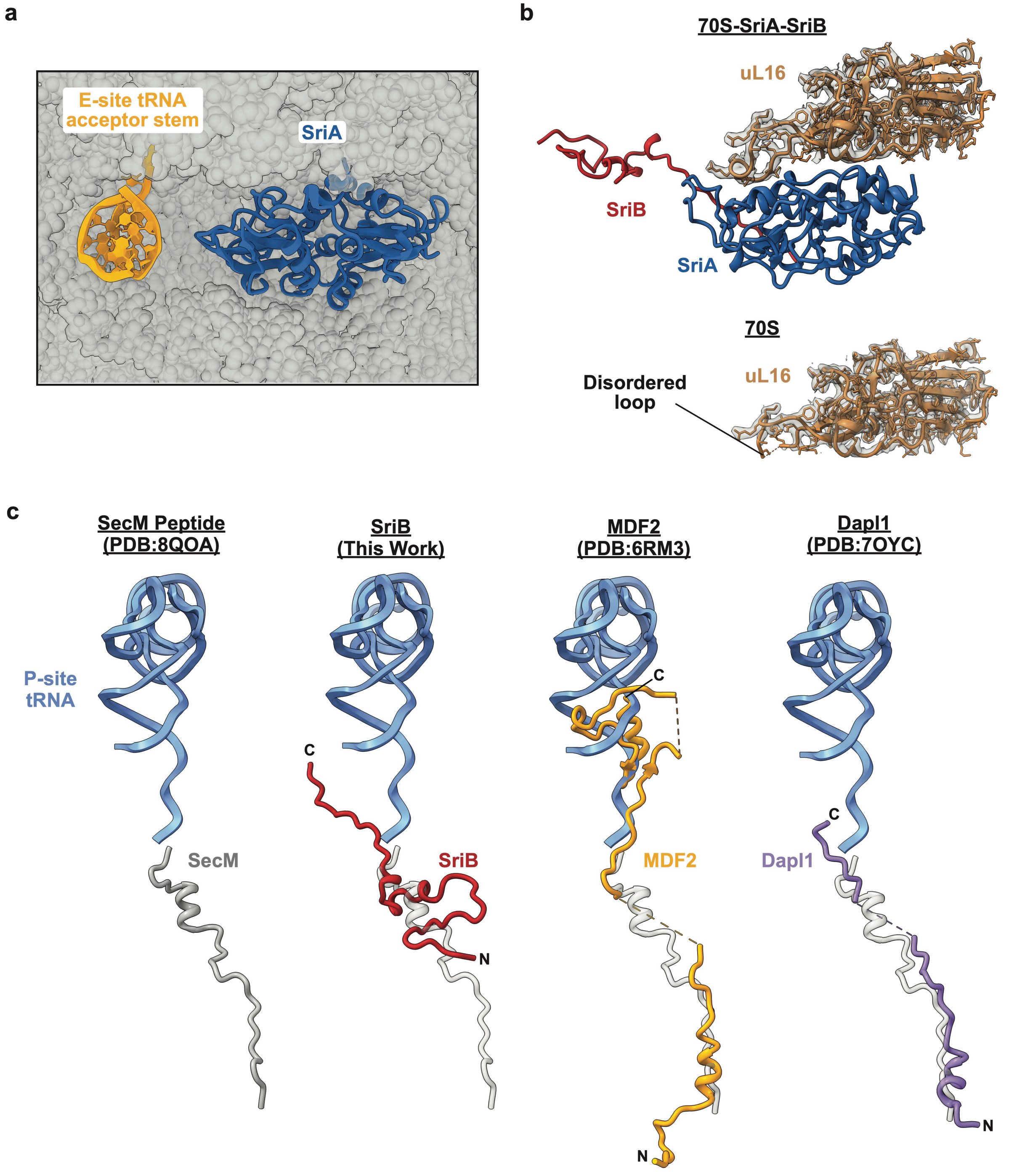

Supplementary Fig. 11: SriA and SriB interactions with the LSU. (a) SriA binding is compatible with the presence of E-site tRNA. (b) Model and cryo-EM density of uL16 in the 70S-SriA-SriB complex (top) compared with the 70S ribosome lacking SriA and SriB (bottom). In the absence of SriA and SriB, a loop of rProtein uL16 is disordered. (c)

Comparison of the SriB N-terminus with a stalled nascent polypeptide chain[4] and hibernation factors MDF2[5] and Dapl1[6], which target the exit tunnel. The SecM peptide is overlaid in grey on the hibernation factors to highlight differences in their paths through the PTC and exit tunnel.

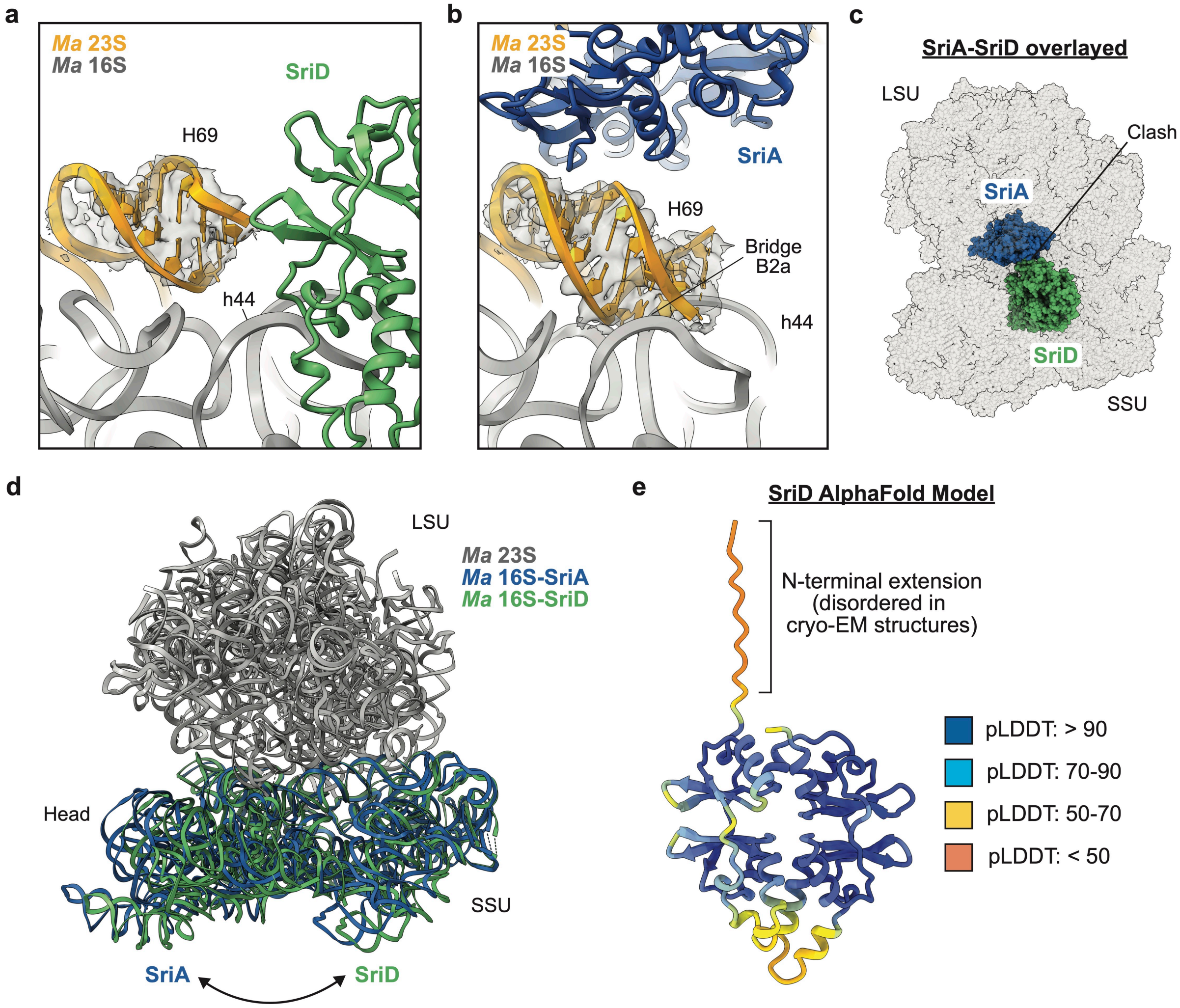

Supplementary Fig. 12: Differences in SriA and SriD binding to the ribosome. (a-b) Conformations of LSU helix H69 and SSU helix h44 in the presence of (a) SriD or (b) SriA. Bridge B2a is formed during SriA binding but is absent in the presence of SriD. (c) Overlay of SriA and SriD binding sites on the ribosome. Simultaneous binding of SriA and SriD would result in a steric clash between the two proteins. (d) *M. acetivorans* ribosomes bound to SriA (blue) and SriD (green) aligned to their 23S rRNA (grey) to highlight differences in the rotational state of the SSU. (e) AlphaFold prediction of SriD colored by confidence score. The SriD N-terminal extension is not resolved in the cryo-EM structure of SriD bound to the *M. acetivorans* ribosome.

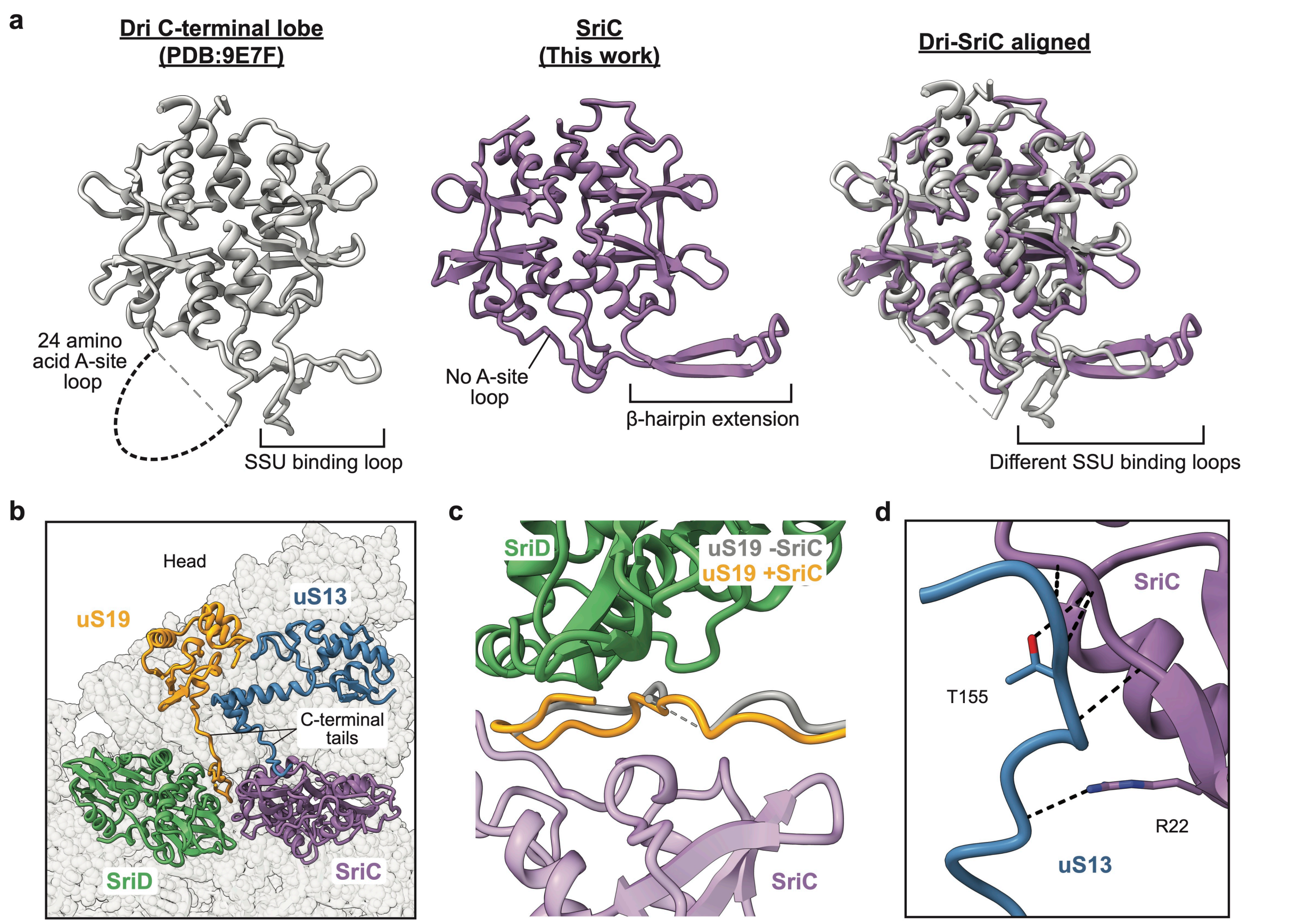

Supplementary Fig. 13: SriC and SriD interactions with the SSU. (a) Comparison of (left) the Dri C-terminal lobe (PDB:9E7F[2]) and (middle) SriC. (right) Alignment of the Dri C-terminal lobe with SriC. (b) Interactions of SriC and SriD with the C-terminal tails of uS13 and uS19. (c) Conformation of the uS19 C-terminal tail in the presence of SriD (grey) or of both SriC and SriD (yellow). (d) Interactions between SriC and the uS13 C-terminal tail.

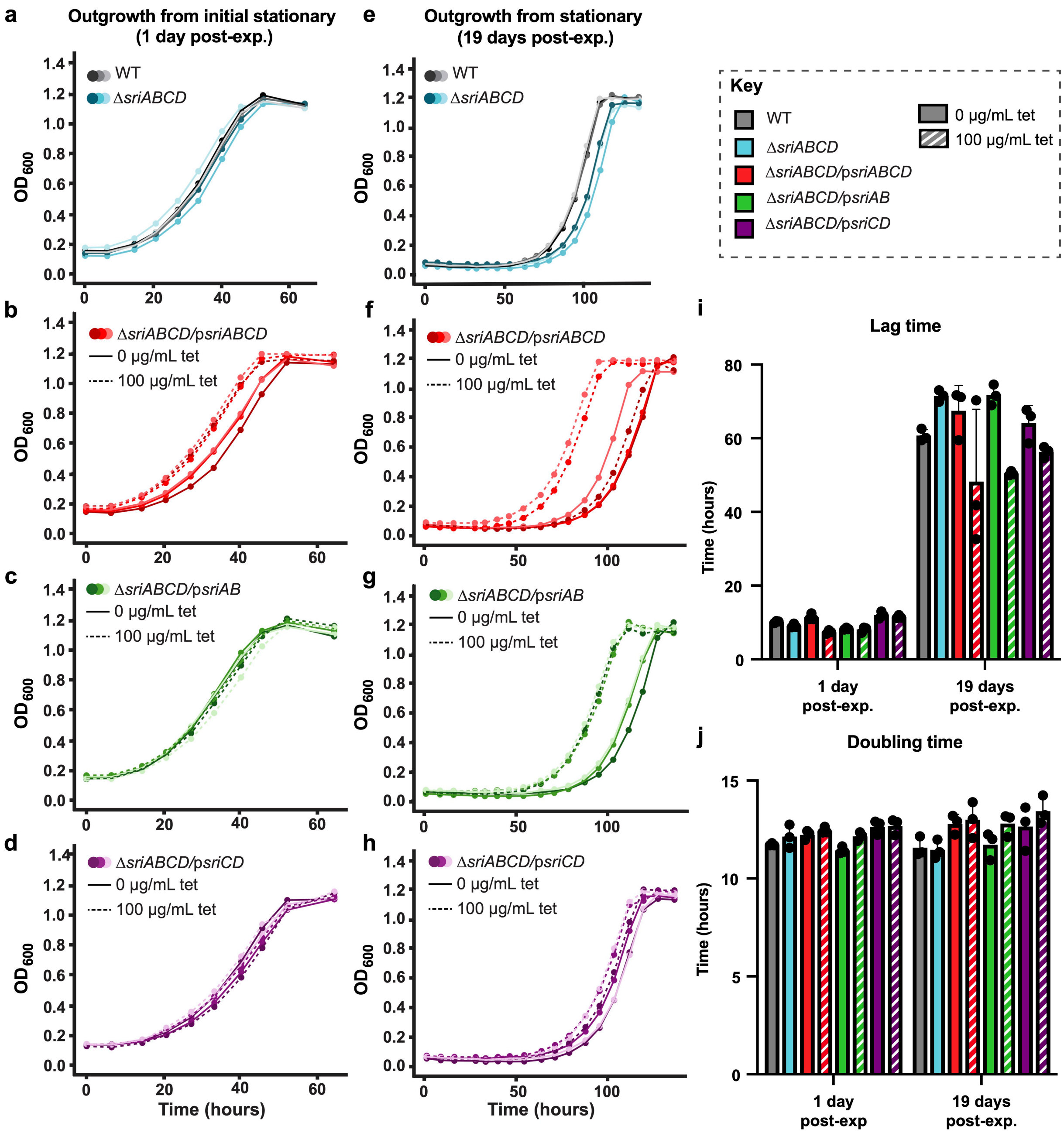

Supplementary Fig. 14: Complementation strain outgrowths from initial stationary and stationary phases. Growth curves of *M. acetivorans* strains outgrown from (a-d) initial stationary (1 day post-exponential) and (e-h) stationary (19 days post-expoonential). Solid lines, 0 µg/mL tetracycline (tet); dashed lines, 100 µg/mL tet. Bar plots of (i) lag times and (j) doubling times per strain. Solid bars, 0 µg/mL tet; striped bars, 100 µg/mL tet. All conditions were cultured in triplicate. Tet was supplemented in outgrowth and

starter culture media. Statistical significance was tested with multiple unpaired Welch's t-tests and reported in Supplementary Table 9 (omitted from figure for visual clarity). Error bars represent standard deviation of triplicates.

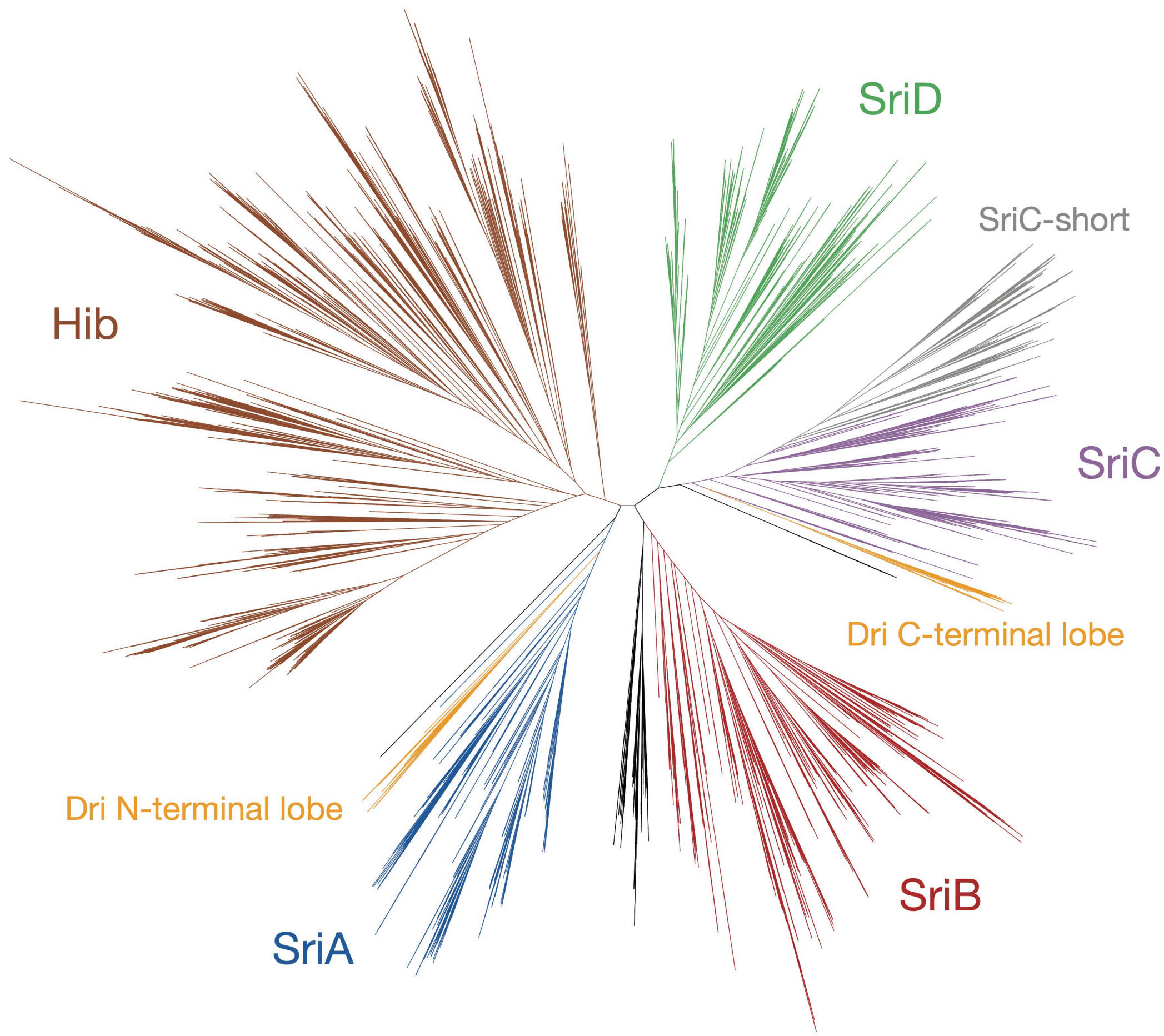

Supplementary Fig. 15: Phylogenetic tree of all Sri proteins and related homologs in archaea, including HibA and Dri (split into N- and C-terminal lobes), mined from proteomes of GTDB representative species. Subclades corresponding to SriA, SriB, SriC, SriD, and HibA were defined as regions of the tree that consistently hit the respective homology model best. SriC-short represents a diversification of SriC proteins predominantly in Nitrososphaerales that have a shorter and less positively charged loop that likely function in a different manner than canonical SriC. Clades colored black do not consistently hit one of the homolog models best and may represent related CBS domain-containing proteins.

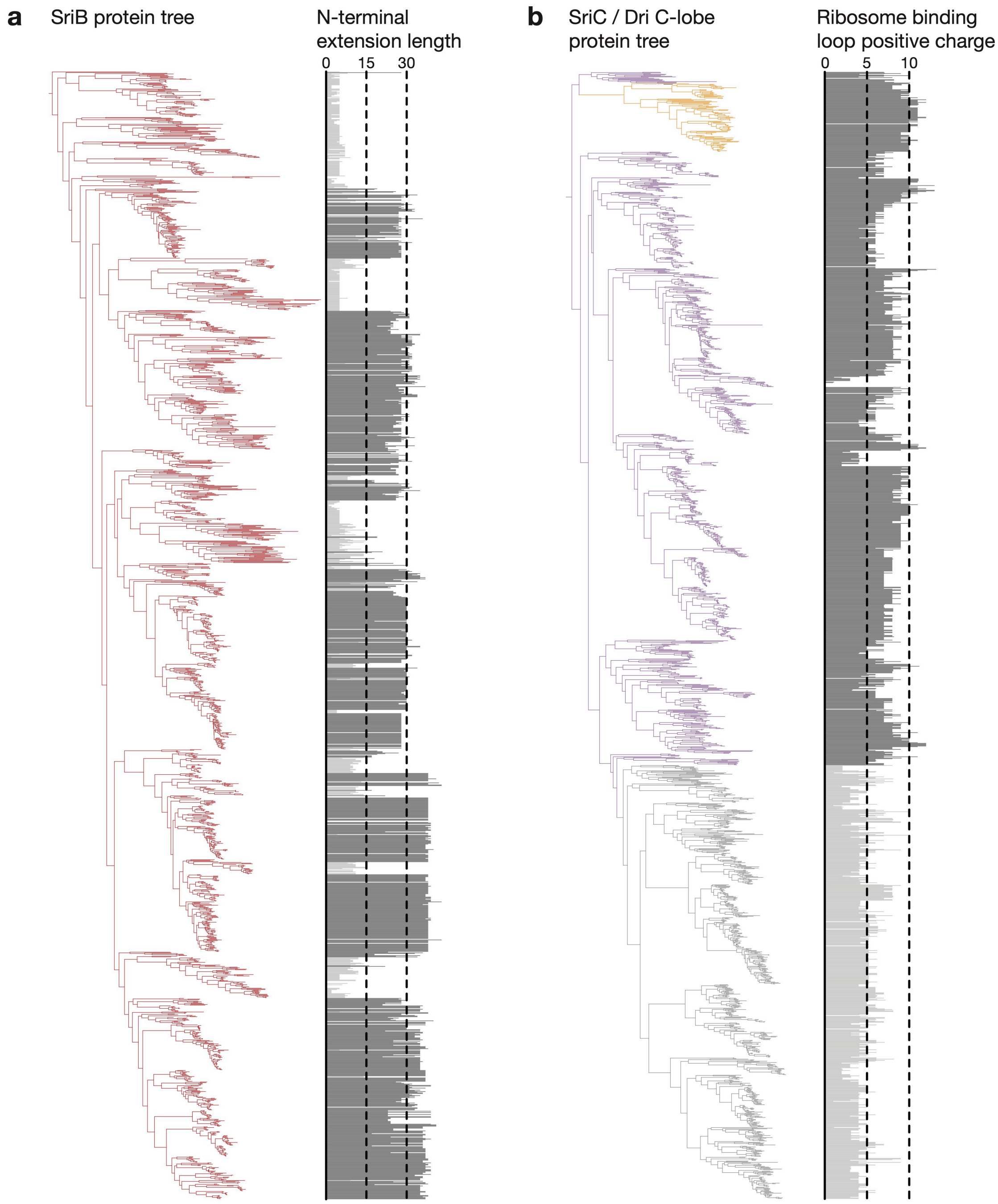

Supplementary Fig. 16: SriB and SriC sections of the Sri-HibA protein tree from Supplementary Fig 15. (a) SriB tree with the length of the N-terminal extension plotted for each homolog. Proteins with an extension of less than or equal to 15 amino acids (colored light grey) were designated SriB-short and were considered separately in co-

occurence, phylogenetic distribution, and conservation analyses. (b) SriC / Dri C-terminal lobe tree with the number of positively charged residues (adjusted for histidine partial charge) of the ribosome binding loop plotted for each homolog. The subclade highlighted in light grey is named SriC-short, a diversification of SriC proteins predominantly in Nitrososphaerales with on average shorter and less positively charged loops. These sequences were considered separately in co-occurrence, phylogenetic distribution, and conservation analyses.

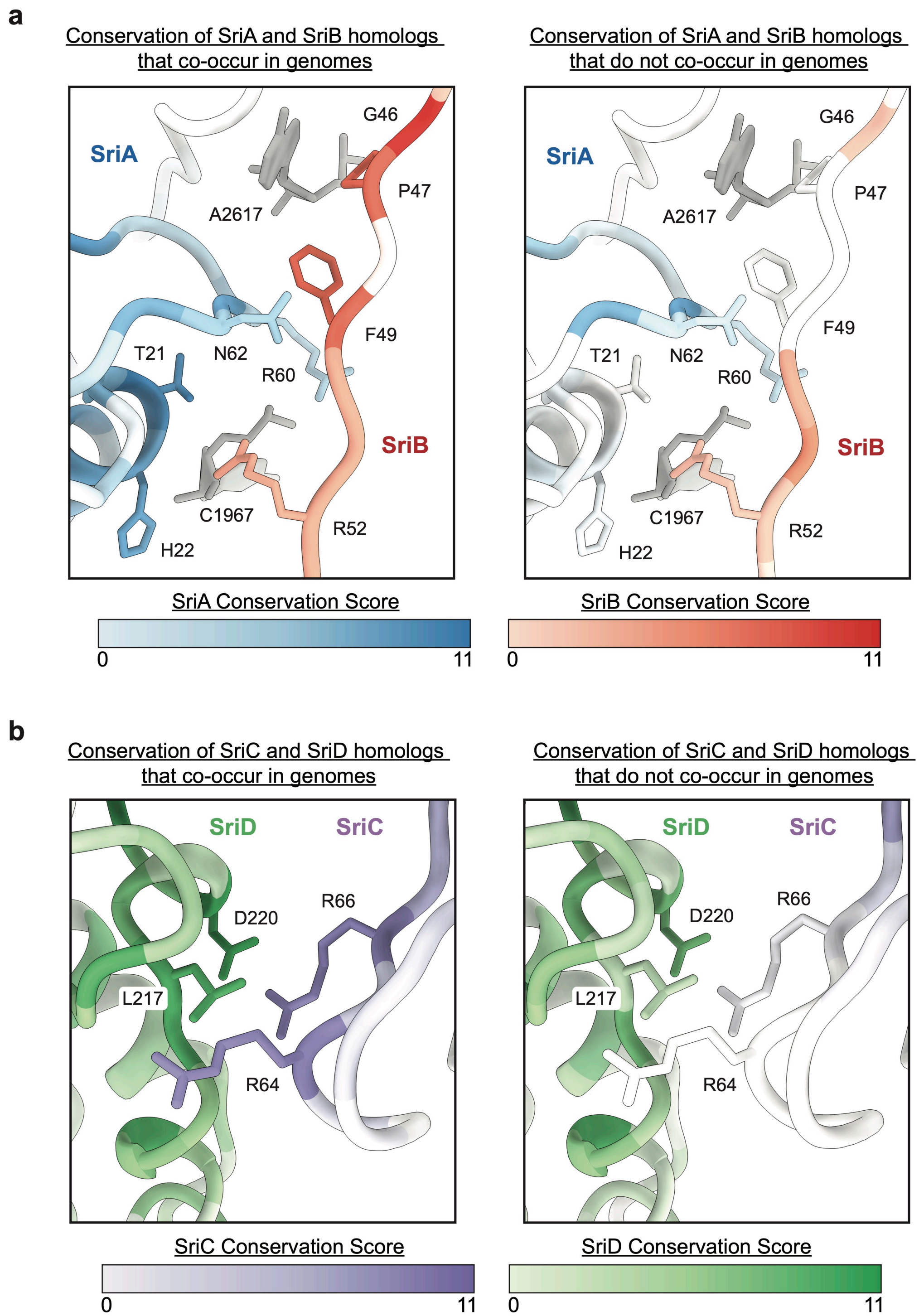

Supplementary Fig. 17: Conservation of Sri-Sri interface residues. (a) SriA-SriB interface residues are colored based on conservation scores from homologs in organisms that encode both proteins (left) or in organisms that have standalone *sriA* or

sriB genes (right). (b) SriC-SriD interface residues are colored based on conservation scores from homologs in organisms that encode both proteins (left) or in organisms that have standalone *sriC* or *sriD* genes (right).

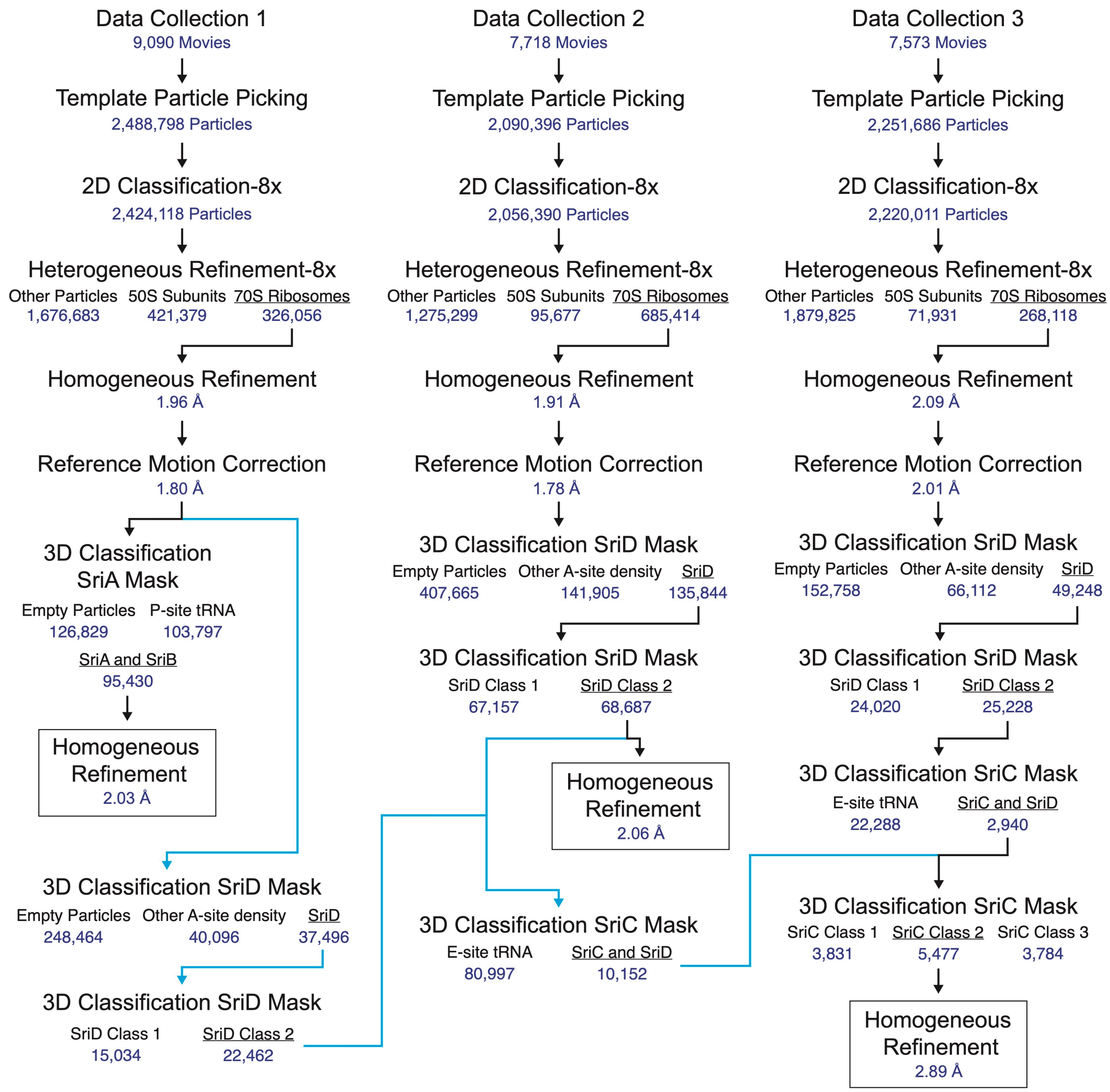

Supplementary Fig. 18: Cryo-EM processing workflow. Blue arrows show the processing path to combine SriD containing-particles from the three datasets for SriC classifications and reconstructions.

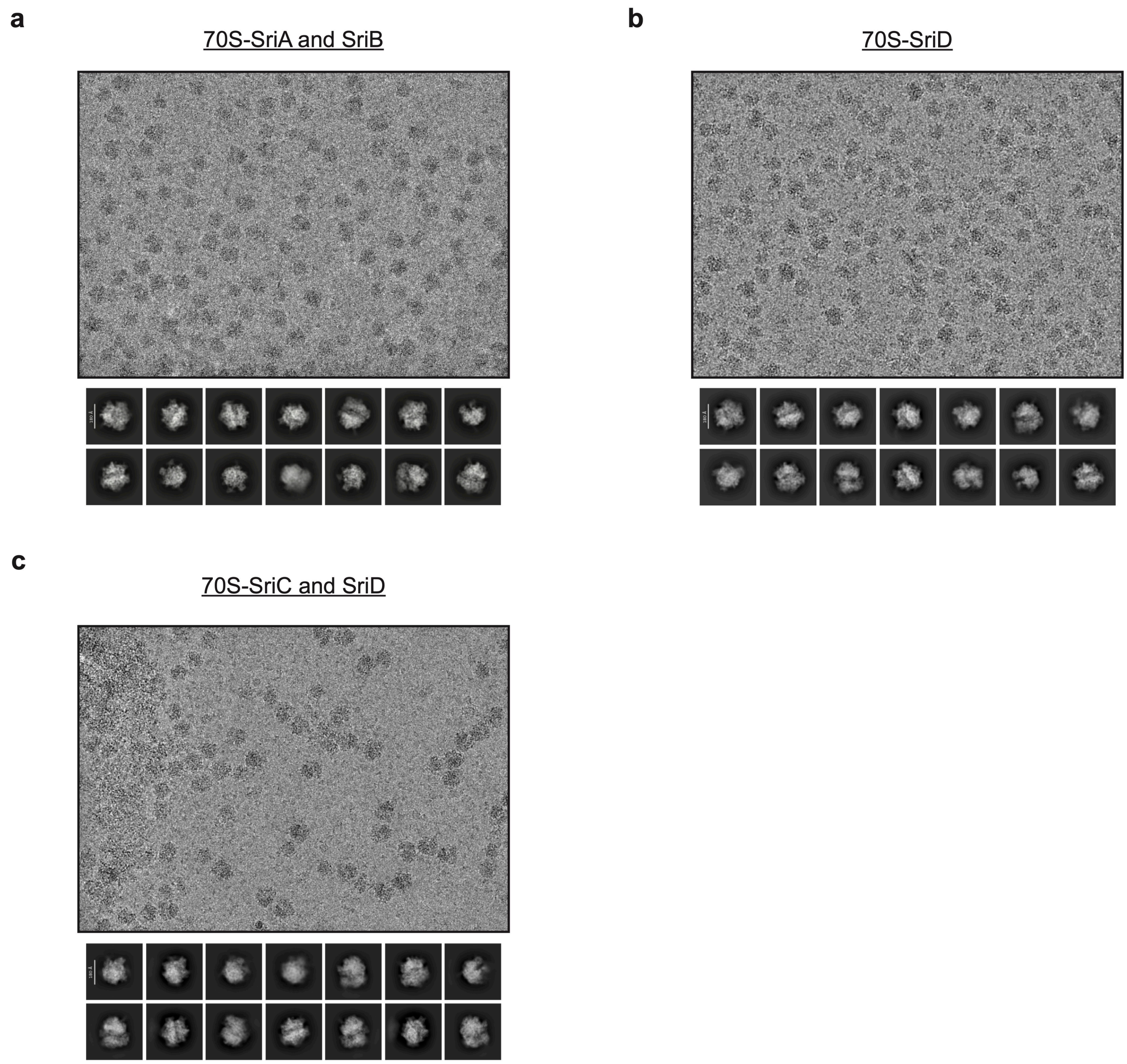

Supplementary Fig. 19: Representative micrographs and 2D class averages for the (a) SriA-SriB, (b) SriD, and (c) SriC-SriD cryo-EM datasets.

Supplementary Table 1. Nucleotide Numbering Conversions

E. coli **23S Numbering**	***M. acetivorans*** **23S Numbering**	***E. coli*** **16S Numbering**	***M. acetivorans*** **16S Numbering**
1942	1967	470	530
1944	1969	1492	1430
2506	2521	1493	1431
2507	2522	1494	1432
2583	2598	1495	1433
2584	2599	1496	1434
2585	2600	1534	1472
2586	2601	1535	1473
2602	2617		

Supplementary Table 2. Primers Used in this Study

**Name**	**Sequence**	**Description**
oMW025	CTCTTTGATACTTACAGGCCACATGTGCTTACA GCATAATTAGGG	CRISPR guide fragment, MA4648-51 target, R
GLC245	CCTTTTTTTTTCGAAGTTTAACAACATCAGTCAC CTAAAAAG	CRISPR guide fragment with pDN201 overhang, F
oMW026	GGCCTGTAAGTATCAAAGAGGTTTTAGAGCTA GAAATAGCAAGTTAAAATAAG	CRISPR guide fragment, MA4648-51 target, F
GLC246	GCCGGCGCGCCTGCAGGTTTTACATGAGGGC TGAAAAGC	CRISPR guide fragment with pDN201 gibson overhang, R
oMW029	GCCCTCATGTAAAACCTGCAGGGTCAATGGTTG ATGAACCTGAG	MA4648-51 upstream homology gibson overhang, F
oMW033	GAAGTTTATTCCTCGATTGTGCTGACATTCATTG TTACTTTTCCTC	MA4648-51_upstream homology gibson overhang, R
oMW034	AAGTAACAATGAATGTCAGCACAATCGAGGAAT AAACTTCAGAGAAAAG	MA4648-51 downstream homology gibson overhang, F
oMW035	TAAGCGGCCGCGATCGCCGGACGAGATTATTG GGAACGATATGC	MA4648-51 downstream homology gibson overhang, R
oMW045	TTTTTTTAAGATTTCTCGATGTTTTTCATTCTG	*sriABCD* (MA4648-51 region) KO genotype primers, F
oMW046	TGTACTTGAATTTCAATTAAGTTACGAGG	*sriABCD* (MA4648-51 region) KO genotype primers, R
oMW047	GTAATTCCAGACCTTGGAGATCC	*SriABCD (*MA4648-51 region) internal KO genotyping primer, R
oMW048	CCTCAATCTGGAAGAAGAACTGG	*sriABCD* (MA4648-51 region) internal KO genotyping primer, F
oMW049	TAAATTAAGGAGGAAATTCAATGAATGTCAGCGA GATAATGTCAG	*sriA* + tetO1 overhang, F
oMW050	ATTATACGAAGTTATCAAGATTATTCCTCGATTG TGTTTATGATATTCC	*sriD* + pJK027A overhang; R
oMW051	ATTATACGAAGTTATCAAGATTAAAGGTCAAACC CTCTCAGG	*sriB* + pJK027A overhang; R
oMW052	TAAATTAAGGAGGAAATTCAATGAATGTGGCGG ACATCATG	*sriC* + tetO1 overhang; F
oMW050	TCACTGAATTTCCTCCTTAATTACTCCGACCACC CGTTG	*sriA* + shine-delgarno + *sriB* overhang; R
oMW051	GTAATTAAGGAGGAAATTCAGTGAACAAATCAG GTAAAAAGGCG	*sriB*, + shine-delgarno + *sriA* overhang; F
oMW052	CCTGAGAGGGTTTGACCTTTAATTAAGGAGGAA ATTCAATGA	*sriB* + shine-delgarno + *sriC* overhang; R

oMW053	TAATTAAGGAGGAAATTCAATGAATGTGGCGGACATCATG	*sriC* + shine-delgarno + *sriB* overhang; F
oMW054	GCATTGAATTTCCTCCTTAATTAGAGCACGGATTTCATGATGTC	*sriC* + shine-delgarno + *sriD overhang*; R
oMW055	CTAATTAAGGAGGAAATTCAATGCAAGTTAAAGACATAATGGTACAAC	*sriD*, shine-delgarno + *sriC* overhang; F
AB90	CTCGTAGAAGGGGAGGTTGCG	*pac* (PurR) (for detection of editing plasmid), F
AB91	ATGACCGAGTACAAGCCCACGG	*pac* (PurR) (for detection of editing plasmid), R

Supplementary Table 3. Plasmids Used in this Study

**Name**	**Description**	**Reference**
pAMG40	E. coli shuttle vector for fosmid retrofitting to enable replication in *M. acetivorans.* Contains pC2A and λattB	[7]
pJK027A	*M. acetivorans* expression vector. Contains PmcrB(tet01).	[7]
pDN201	pJK027A derived. Contains PmcrB(tet01)::Spy *cas9*	[8]
pMW001	Gibson assembly of MA4648-50 guide 1 fragment + pDN201 (PmeI)	This study
pMW003	Gibson assembly of pMW001 (AscI) + MA4648-51 homology fragment	This study
pMW005	Co-integrant of pMW003 + pAMG40	This study
pMW007	pJK027A (HindIII, NdeI) + *sriABCD* genomic fragment	This study
pMW008	pJK027A (HindIII, NdeI) + *sriAB* genomic fragment	This study
pMW009	pJK027A (HindIII, NdeI) + *sriCD* genomic fragment	This study
pMW010	Cointegrate of pMW007 + pAMG40	This study
pMW011	Cointegrate of pMW008 + pAMG40	This study
pMW012	Cointegrate of pMW009 + pAMG40	This study

Supplementary Table 4. Strains Used in this Study

**Strain**	**Genotype**	**Reference**
WWM60	*M. acetivorans* ∆*hpt*:: *PmcrB-tetR*	[7]
DDN482	WWM60 ∆*sriABCD* (MA4648-4651)	This study
DDN483	WWM60/pMW010 (Ptet01-*sriABCD*)	This study
DDN484	WWM60/pMW011 (Ptet01-*sriAB*)	This study
DDN485	WWM60/pMW012 (Ptet01-*sriCD*)	This study
DDN486	DDN482/ pMW010 (∆sriABCD + Ptet01-*sriABCD*)	This study
DDN487	DDN482/ pMW011 (∆sriABCD + Ptet01-*sriAB*)	This study
DDN488	DDN482/ pMW012 (∆sriABCD + Ptet01-*sriCD*)	This study

Supplementary Table 5. Protein Sequences Used for Recombinant Expression in this Study

Name	Sequence
SriA	MNVSEIMSEGPVSIKERDFVTHARQLMRDYLFRSLVVVDEGNRLVGMLNDQDIMRVTS TRSNVTVGGYARPSPTVTPDMDVVKAAKLMVQSKQNRVPVVKSTTDHTVVGVLSDVDI LRNAELPRSASKTIDMVMTKKVKTCSPDERISKVWNYMTETDYTGIPVVSKKGDPIGMI TRRDIIKAGILRMSIEDERAARPNESPKVEKIMSTPAYTLSENDSVKSAIEMIIQHDIGRVT IVNEQGKISGIADRQDLMNAFVNGWSEHHHHHH
SriB	MILLNKNTTFVPVEKMNVQPQVNKSGKKAQQKDPHSVSSMGTMRIGPSFKSRIAEHEG KILALATRDVVTLPPTAAIMEAVRIMTERRFRRIPITDAGTGRLEGVVTSVDIIDFLGGGS RNLLVENRFKGNLLAAINEEVRQIMQTDVAYLNDQADFKDAVTTMLERRTGGLPIVNDE MQVIAIFTERNAVELMGGIVTNKTVDEYMTKNVTMVTTDTPIGQAAKVMVQNRFRRLPV VKDGIFAGIVTASDIVHFLGRGDAFSKLTTGNIHEALDQPVGSIVSQELIWTSPGTDMGK AMEIMLEKKIGSLPVLEDGMLRGIITESDFLRGFDLHHHHHH
SriC	MNVADIMSSPVYAINIDEPVSRARKLMLRHRISTLLVLNEGKMVGIVTKSDISNRLAQAE PLWRRRPIDQIPIKLLMTESVITIYPEASISQAAALMLENGVHDIPVVKNDIVGIVTRTDIVR YVAEHADEIDTKISTLMTDDIVSVHRHHTINHVIEEMNKNEIERVIVKDDAGKPVGVISKR NLALNLLTDNEGKLSTKSIKMARKSSPGGQKTYRYVKEVPLTAEDIMITPIISIDVNEKISI AAKKLIEEEITALPVSDGEEIVGILSRTDIMKSVLHHHHHH
SriD	MQRIETKTLDTHEVKCMQVKDIMVQPHKIDKSDTISHALDLMEKKDTKRLLVVHDNQVL GVLTMRGLTEQLGTRRKQSKPASSLHVATAVSDNFVKVLPDTDVKDALTLMKKKGGVII VTDNGNAMGWVTPQELMKVNHFTGFAGEVMEKNPIIVSPSDRVSHARRLILDKNVGRL PVIENGKLVGIIAEDDIAFAMRSFRDLVADNQQDSRIKNLLVGDIMTRSVVNVYTNTPLS DTVDTMLEYDVGGVPVLNLEEELVGFLARRNIINTIEEHHHHHH

Supplementary Table 6. NLuc *In Vitro* Translation Reporter mRNA Sequence

The SD sequence is **bolded**; The start codon is <u>underlined</u>

Name	Sequence
NLuc mRNA	GGUCCCAAAGUGAUUUAAUAAAUU**AAAGGAGGA**AAUUAAA<u>AUG</u>GUCUUCACACU<br>CGAAGAUUUCGUUGGGGACUGGCGACAGACAGCCGGCUACAACCUGGACCAAGU<br>CCUUGAACAGGGAGGUGUGUCCAGUUUGUUUCAGAAUCUCGGGGUGUCCGUAA<br>CUCCGAUCCAAAGGAUUGUCCUGAGCGGUGAAAAUGGGCUGAAGAUCGACAUCC<br>AUGUCAUCAUCCCGUAUGAAGGUCUGAGCGGCGACCAAAUGGGCCAGAUCGAAA<br>AAAUUUUUAAGGUGGUGUACCCUGUGGAUGAUCAUCACUUUAAGGUGAUCCUGC<br>ACUAUGGCACACUGGUAAUCGACGGGGUUACGCCGAACAUGAUCGACUAUUUCG<br>GACGGCCGUAUGAAGGCAUCGCCGUGUUCGACGGCAAAAAGAUCACUGUAACAG<br>GGACCCUGUGGAACGGCAACAAAAUUAUCGACGAGCGCCUGAUCAACCCCGACG<br>GCUCCCUGCUGUUCCGAGUAACCAUCAACGGAGUGACCGGCUGGCGGCUGUGC<br>GAACGCAUUCUGGCGUAAGGAUCCGGAGAGCUCCCAACGCGUUGGAUGCAUAG<br>CUUGAGUAUUC

Supplementary Table 7. Cryo-EM Data Collection and Processing

**Reconstruction**	**70S-SriA-SriB**	**70S-SriD**	**70S-SriC-SriD**
Magnification	105,000	105,000	105,000
Voltage (kV)	300	300	300
Electron Exposure ($e^-/Å^2$)	40	40	40
Defocus Range (μm)	-0.5/-1.5	-0.5/-1.5	-0.5/-1.5
Pixel Size (Å)	0.8264	0.8264	0.8264
Symmetry Imposed	C1	C1	C1
Initial Particle Images	2,488,798	2,090,390	6,830,880
Final Particle Images	95,430	68,687	5,477
Map Resolution (Å)	2.03	2.06	2.89
FSC Threshold	0.143	0.143	0.143

Supplementary Table 8. Model Refinement Statistics

Model	**70S-SriA-SriB**	**70S-SriD**	**70S-SriC-SriD**
Model component			
Model resolution (Å)	2.11/2.90 (50S/30S)	2.07/2.48 (50S/30S)	2.89/3.06 (50S/30S)
FSC threshold	0.5	0.5	0.5
Map sharpening *B* factor ($Å^2$)	35.5/48.6 (50S/30S)	30.7/48.3 (50S/30S)	25.1/27.9 (50S/30S)
Model composition			
Non-hydrogen atoms	157707	159420	150576
Mg^{2+} ions	305	314	314
Waters	8740	10476	0
Mean *B* Factors ($Å^2$)			
RNA	51.58	49.86	44.19
Protein	62.40	60.01	51.40
Waters	50.41	48.73	-
Other	58.21	56.95	33.33
R.m.s. deviations from ideal values			
Bond (Å)	0.005	0.005	0.006
Angle (°)	0.655	0.669	0.843
Molprobity score	1.86	1.89	2.26
Clash Score	7.91	7.50	8.60
Rotamer outliers (%)	2.59	2.82	5.04
Ramachandran plot			
Favored (%)	97.39	97.28	96.14
Allowed (%)	2.50	2.68	3.77
Outliers (%)	0.11	0.04	0.09
RNA validation			
Angles outliers (%)	0.01	0.01	0.01
Sugar pucker outliers (%)	0.9	0.7	0.7
Average suiteness	0.634	0.633	0.596

Supplementary Table 9. Lag duration of each condition tested against Δ*sriABCD* of respective outgrowth; results of multiple two-tailed, unpaired t-tests with Welch correction.

Outgrowth	Condition	P-value
**1 day post-exp.**		
	WT (WWM60)	0.053229
	Δ*sriABCD*/p*sriABCD*; 0 µg/mL tet	0.02738 #
	Δ*sriABCD*/p*sriABCD*; 100 µg/mL tet	0.01775
	Δ*sriABCD*/p*sriAB*; 0 µg/mL tet	0.121942
	Δ*sriABCD*/p*sriAB*; 100 µg/mL tet	0.144763
	Δ*sriABCD*/p*sriCD*; 0 µg/mL tet	0.016621 #
	Δ*sriABCD*/p*sriCD*; 100 µg/mL tet	0.004568 #
**19 days post-exp.**		
	WT (WWM60)	0.001196
	Δ*sriABCD*/p*sriABCD*; 0 µg/mL tet	0.419319
	Δ*sriABCD*/p*sriABCD*; 100 µg/mL tet	0.176093
	Δ*sriABCD*/p*sriAB*; 0 µg/mL tet	0.920538
	Δ*sriABCD*/p*sriAB*; 100 µg/mL tet	0.000477
	Δ*sriABCD*/p*sriCD*; 0 µg/mL tet	0.106643
	Δ*sriABCD*/p*sriCD*; 100 µg/mL tet	0.000267
**28 days post-exp.**		
	WT (WWM60)	0.056055
	Δ*sriABCD*/p*sriABCD*; 0 µg/mL tet	0.824674
	Δ*sriABCD*/p*sriABCD*; 100 µg/mL tet	0.011197

	Δ*sriABCD/*p*sriAB*; 0 µg/mL tet	0.769018
	Δ*sriABCD/*p*sriAB*; 100 µg/mL tet	0.007458
	Δ*sriABCD/*p*sriCD*; 0 µg/mL tet	0.5945
	Δ*sriABCD/*p*sriCD*; 100 µg/mL tet	0.17669

[#] average lag longer than Δ*sriABCD*

Supplementary References

1. Deatherage, D. E. & Barrick, J. E. Identification of Mutations in Laboratory-Evolved Microbes from Next-Generation Sequencing Data Using breseq. in *Engineering and Analyzing Multicellular Systems* (eds Sun, L. & Shou, W.) vol. 1151 165–188 (Springer New York, New York, NY, 2014).
2. Nissley, A. J. *et al.* Structure of an archaeal ribosome reveals a divergent active site and hibernation factor. *Nat Microbiol* **10**, 1940–1953 (2025).
3. Jumper, J. *et al.* Highly accurate protein structure prediction with AlphaFold. *Nature* **596**, 583–589 (2021).
4. Gersteuer, F. *et al.* The SecM arrest peptide traps a pre-peptide bond formation state of the ribosome. *Nat Commun* **15**, 2431 (2024).
5. Barandun, J., Hunziker, M., Vossbrinck, C. R. & Klinge, S. Evolutionary compaction and adaptation visualized by the structure of the dormant microsporidian ribosome. *Nat Microbiol* **4**, 1798–1804 (2019).
6. Leesch, F. *et al.* A molecular network of conserved factors keeps ribosomes dormant in the egg. *Nature* **613**, 712–720 (2023).
7. Guss, A. M., Rother, M., Zhang, J. K., Kulkarni, G. & Metcalf, W. W. New methods for tightly regulated gene expression and highly efficient chromosomal integration of cloned genes for Methanosarcina species. *Archaea* **2**, 193–203 (2008).
8. Nayak, D. D. & Metcalf, W. W. Cas9-mediated genome editing in the methanogenic archaeon *Methanosarcina acetivorans*. *Proc. Natl. Acad. Sci. U.S.A.* **114**, 2976–2981 (2017).
