# Supplementary material for: A family of archaeal hibernation factors that bind in tandem and protect ribosomes in dormant cells
Source: Nat Commun. 2026 Jun 25;17:8083. doi: 10.1038/s41467-026-74727-0 (PMC13454478; doi:10.1038/s41467-026-74727-0)
Supplement: Supplementary file 4 [file 41467_2026_74727_MOESM2_ESM.pdf]

Description of Additional Supplementary Files:

Supplementary Data 1: RNA sequencing coverage

Supplementary Data 2: Distribution of aS36 homologs

Supplementary Data 3: Sequences of aS36 homologs

Supplementary Data 4: Distribution of Sri and HibA homologs

Supplementary Data 5: Sequences of SriA homologs

Supplementary Data 6: Sequences of SriB homologs

Supplementary Data 7: Sequences of SriC homologs

Supplementary Data 8: Sequences of SriD homologs

Supplementary Data 9: Sequences of archaeal HibA homologs

S**upplementary Data 10:** Sequences of bacterial HibA homologs
